# A novel phased-array thermography concept for non-destructive testing

**DOI:** 10.1038/s41598-025-26380-8

**Published:** 2025-11-07

**Authors:** Cristiano Martinelli, Michele Meo

**Affiliations:** Department of Aeronautics and Astronautics, University of Southampton, Southampton, SO16 7QF UK

**Keywords:** Non-destructive testing, Thermography, Aerospace engineering, Defect identification, Engineering, Mathematics and computing

## Abstract

Phased array technology involves the coordinated control of multiple elements to steer and focus elastic, electromagnetic, light, seismic, and radio waves in a specific location or direction. In structural integrity applications, it enables the precise inspection of materials and the identification of flaws/defects in structures. In this paper, we proposes a novel phased array method based on the steering and focusing of thermal waves, not previously explored for applications in NDT, named Phased-Array Thermography (PAT). This new three-dimensional approach aims to overcome the main limitations of most of the Active Infrared Thermography (IRT) methods that uniformly heat the component surface and generate a normal temperature gradient, resulting in lack of control in the gradient direction and, ultimately, limiting the identification capabilities of IRT. PAT leverages an array of heating elements to precisely steer and control the thermal wavefront. A closed-form analytical solution of the thermal wave propagation is derived and validated against numerical simulations. Then, the accuracy of proposed method is assessed via thermal Finite Element (FE) simulations of an aluminium plate by comparing PAT with a commonly used IRT technique such as the Pulsed Thermography (PT). Finally, experimental analyses of an aluminium plate with flat bottom holes and a composite plate with impact damage are performed to validate the proposed methodology. This novel approach to thermal wavefront steering via phased array technology introduces a previously unexplored mechanism for controlled heat wavefront, with transformative potential for non-destructive evaluation, structural health monitoring, and adaptive manufacturing systems.

## Introduction

Non-Destructive Testing (NDT) techniques are fundamental for evaluating the structural integrity of mechanical and aerospace components. Several NDT techniques, such as Eddy-Current (EC)^1^, Infrared Thermography (IRT)^2^, Ultrasonic Arrays (UA)^3^, Radiography^4^, and Magnetic Particle Inspection (MPI)^5^ have been developed and successfully applied to assess the presence of defects in aerospace components. Due to its speed, reliability, and ability to evaluate complex structures, IRT is particularly suitable for the evaluation of structural integrity of aerospace components such as the primary and secondary structure of aircraft and rotorcraft^6–8^. IRT is distinguished into active and passive thermography; passive IRT leverages the natural thermal fluctuations of components to evaluate and monitor their structural integrity. Such condition can be induced by the environment, e.g. by the daily change in temperature induced by the sunlight^9^, or by the deformation of the structure, e.g. static and dynamic mechanical loading^10,11^. In contrast, active IRT uses heating elements or flash lamps to heat the tested component and evaluate the presence of surface and subsurface defects. Several active IRT techniques have been proposed in the literature: Ciampa et al.^2^ classified them based on the active heating method, considering four physical principles: optical radiation, ultrasonic wave propagation, electromagnetic radiation, and material-enabled radiation. One of the most widely used optical radiation active IRT techniques is the Pulsed Thermography (PT)^12^. PT applies short high-power external thermal energy via lamps to heat the surface of the tested component. The thermal response of the stimulated surface is then monitored via an infrared thermal (IR) camera. Damaged regions and subsurface defects reduce the thermal conduction of the component, thus, they can be identified by temperature anomalies and differences with respect to the undamaged regions. PT is widely used in engineering and several variation exist in the literature, including Lock-in Thermography^13^ and Long Pulse Thermography (LPT)^14–16^. The advantages of PT are the non-contact nature and the speed of the procedure. Nonetheless, this technique presents a series of disadvantages: the reflected heat of the components under evaluation can interfere with the measurement, generating noise and abnormal thermal patters, deep defects, located far from the surface, are difficult to evaluate (within few millimetres from the heated surface^2^), and cracks perpendicular to stimulated surface cannot be identified because they do not interact with the thermal gradient. IRT can also be performed via ultrasonic wave propagation^17,18^: this approach, generally named Ultrasonic Stimulated Thermography (UST), uses transducers (e.g. piezoelectric transducers) to dynamically excite the component under evaluation, causing the motion of contiguous surfaces of cracks. The friction induced by the relative motion of the crack surfaces generates a local increase of temperature that can be monitored and evaluated with a high resolution thermal camera. The main advantages of UST is the capability of identifying micro-cracks and cracks oriented in different directions. UST also has some disadvantages and limitations: in-depth defects are difficult to identify as the technique is limited by penetration capacity of ultrasonic waves. Additionally, it requires an effective coupling between the excitation source and the evaluated component. Other active IRT approaches are based on electromagnetic radiation. Among these, Eddy Current Stimulated Thermography (ECST) is one of the most utilised techniques. ECST uses electromagnetic radiation to induce eddy currents on components made of conductive material. The currents generate localised heating in the material via the Joule effect which is uniform in defect-free areas. The presence of cracks and defects is perceived by the eddy currents as a discontinuity which generates anomalies in the distribution of heat. This can be recorded with the aid of an IR camera, identifying the location of the defects/cracks. The ECST has the advantage of being a fast, accurate, and non-contact NDT. Although the sensitivity of ECST decreases with depth, the technique is able to identify in-depth defects (e.g. 50 mm in CFRP when the electromagnetic excitation is set at 100 kHz^19^) . Nonetheless, like most IRT techniques, it suffer from environmental noise from external thermal sources and requires conductive material to generate eddy currents and the Joule heating effect. IRT can also be achieved via material-embedded radiation: the idea is to generate heat internally in the component rather than relying on external sources like lamps for PT, transducers for UST, and electromagnetic induction coil in ECST. This approach is particularly interesting for aerospace applications as it exploits embedded elements to induce thermal gradients in the tested components without the need of external heating sources, enabling rapid and cost-effective evaluations of the structural integrity of the mechanical components. Ciampa et al.^2^ distinguished the material-embedded thermography into two sub-categories: Direct Material Based Thermography (DMT) and Indirect Material Based Thermography (IMT). The latter requires the implementation of thermo-resistive elements in the evaluated component, during the manufacturing process. Such elements are then used as heating sources during the evaluation of the integrity of the components. Several examples of IMT are available from the literature. For example, Ahmed et al.^20^ proposed to embed a heat emitting layer in composite material to perform enhanced non-destructive evaluation of the components. De Villoria et al.^21^ proposed to embed electrically conductive Carbon Nanotubes (CNTs) in composite material to simultaneously increase the mechanical properties of the composite material and achieve IMT via electrical contact. The authors demonstrated via experimental tests that the proposed approach requires low power and it is able to identify even small defects and cracks. Other works that utilised embedded material to perform IMT are available at the following references^22–24^.

All the previously described active IRT techniques are based on simultaneous activation of all the internal/external heating elements/sources to generate a thermal gradient and identify defects within the component. Nonetheless, the simultaneous activation of all the heating elements generate a single well-defined thermal wave that is normal to component surface and ultimately limits the identification and detection of defects, especially those that are not parallel to component surface. On the contrary, Phased Arrays (PA) systems utilise spatially distributed transducers to generate and read signals or waves with different phases. This practice reinforces the generated signal which can be focused or steered towards the desired location. The phased-array principle has been extensively used in engineering and technology: for example phased array systems are used in antennas for wireless communications^25^ to improve millimetre-wave signals, in radar for air traffic control, military defence systems, and space-borne surveillance^26^ to locate and identify the presence of aircraft, ships, satellites, and ballistic missiles, in ultrasonic image generation for medical purposes^27^, in ultrasonic arrays and in elastic wave scattering for NDT^3,28,29^ and structural health monitoring^30^ of mechanical and aerospace components. In this paper, we present a novel active NDT method for detecting defects and damages in aerospace and mechanical components. The method, named Phased Array Thermography (PAT) combines the features of PA and IRT and exploits an array of independently controlled embedded heating elements for generating well-defined thermal waves. Inspired by phased-array systems, the heating elements are arranged on the bottom surface of the evaluated component to form a thermal array. The array generates the desired thermal wave, controlling the resulting thermal gradient and wave front which can be steered and focused on a specific location of the evaluated component. Firstly, an analytical formulation for the wave front propagation is presented and validated against FE simulations. The derived analytical solution can determine thermal wave fronts in the investigated component without the need of computationally expensive and time-consuming simulations, resulting in an efficient design tool. The feasibility of the proposed PAT method is assessed through Finite Element Analysis (FEA), which confirms its ability to generate and steer thermal wavefronts effectively. Then, experimental validation is carried out through laboratory experiments on aluminium and composite plates containing known defects. Comparative numerical and experimental analyses against the conventional PT method are also performed to evaluate the effectiveness of the proposed method. The results demonstrate that the proposed PAT results in a fast, accurate, and reliable IRT technique, capable of identifying defects and cracks that are undetected with the classical PT approach.

## Methodology and background theory

**Fig. 1:**
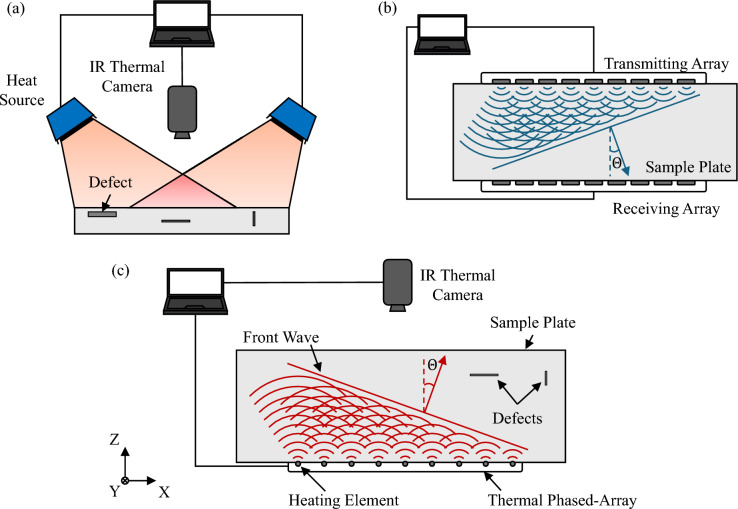
Schematic representation of Pulsed Thermography (PT) (**a**) and Ultrasonic Arrays (UA) (**b**) and Phased-Array Thermography (PAT) (**c**).

The proposed PAT method builds on key-concepts from PT, PA, and UA which are graphically illustrated in Fig. 1a–b. PT uses heat sources, typically lamps, to uniformly increase the surface temperature of the tested component. Then, the heat source is switched off, and the thermal transient response is monitored to identify defects. Such defects have a lower heat conductivity than the undamaged areas, and thus the temperature gradients become visible during the cooling phase, locating defects. UA uses a different approach to identify defects and cracks in solid components: ultrasonic waves are generated by a emitter array, which is constituted by several transducers, and a receiver array is used to receive the generated ultrasonic wave. Most UAs use two operational modes: the first, named back wall reflection (BWR)^31^ and the through-transmission method (TTM)^31^. In both cases, the arrays can fire the transducers with a certain time delay that allows the steering and focusing of the resulting wave beam^3^. In this way, the ultrasonic wave can be directed towards a specific direction/location of the component, enabling the inspection of larger portions of material with efficient scanning. The proposed PAT applies the concept of phased arrays to IRT: the approach is graphically illustrated in Fig. 1c: similarly to UA, PAT exploits a phased-array which consists of multiple heating elements. The figure shows that firing each element with a certain time delay enables the control of the thermal wave, generating the desired wave front which can be steered or focused in a specific location of the component.

The temperature of the opposite surface of the components is recorded via IR camera and post-processing is performed to identify defects. Using this approach, unlike PT or the previously mentioned IMT, the directed thermal wave can potentially interact with detects that are oriented in any direction, enhancing the detection capabilities and IRT.

## Analytical and numerical models for thermal wave front prediction

This section presents an analytical closed-form solution and the associated FE models for the computation of the wave front of the thermal wave generated by thermal phased-arrays. To obtain a simplified model, only a vertical section of the component is considered and heating elements are modelled as point heat sources.

### Analytical model

The analytical solution of the problem is obtained by solving the fundamental partial differential equation of heat diffusion:

1 $$\begin{aligned} \frac{\partial T(\textbf{x},t)}{\partial t} = \alpha \nabla ^2 T(\textbf{x},t) + \frac{\Phi }{\rho c_p} \delta (\textbf{x}) \end{aligned}$$

where $T(\textbf{x},t)$ is the temperature of the considered body, *t* represents the time, $\textbf{x}$ is spatial vector constituted of directions X, Y, and Z, $c_p$ is the specific heat of the material, $\rho$ is the density of the material, $\alpha$ is diffusivity of the material which can be computed as $\alpha = k/(\rho c_p)$, *k* is the material conductivity, $\phi$ is a constant heat flux that is applied to system, and $\delta (\textbf{x})$ is the delta Dirac and represents a specific point location where the heat source is applied. Eq. 1 represents the parabolic model for heat conduction: this model is widely used in engineering contexts^16,32^ and represents an accurate modelling of heat transmission in practical applications^33^. The homogenous version of Eq. 1 for a 2D problem reads:

2 $$\begin{aligned} \frac{\partial T(x,y,t)}{\partial t} = \alpha \left( \frac{\partial ^2 T(x,y,t)}{\partial x^2} + \frac{\partial ^2 T(x,y,t)}{\partial y^2} \right) \end{aligned}$$

Considering an infinite 2D plate, the solution for an instantaneous point source which liberates the unitary temperature concentration $q = 1$ k/m$^2$ at $t = 0$ s has the following well-known form^34,35^:

3 $$\begin{aligned} T(x,y,t) - T_0= \frac{1}{4 \pi \alpha t} e^{-\frac{(x-x_0)^2+ (y-y_0)^2}{4\alpha t}} = \frac{1}{4 \pi \alpha t} e^{-\frac{r^2}{4\alpha t}} \end{aligned}$$

where $r^2 = (x-x_0)^2+(y-y_0)^2$ and $T_0$ is the room temperature. Eq. 3 represents the analytical solution for an instantaneous point heat source, i.e. for a forcing function of the type $\frac{\Phi }{\rho c_p} \delta (\textbf{x})\delta (t)$. To obtain the solution of Eq. 1, where a continuous point source is applied, the solution of Eq. 3 must be convoluted over time *t*, using the following integral:

4 $$\begin{aligned} T(x,y,t) = T_0+\frac{\Phi }{\rho c_p}\int ^{t^*}_{0} \frac{1}{4 \pi \alpha t}e^{-\frac{r^2}{4\alpha t}} dt = T_0+\frac{\Phi }{4 \pi \alpha \rho c_p}\int ^{t^*}_{0} \frac{1}{t}e^{-\frac{r^2}{4\alpha t}} dt \end{aligned}$$

where $t^*$ represents the time at which we want to know the temperature distribution. The integral of Eq. 4 can be solved by applying a change of integration variable. We introduce *v* as new integration variable and we state the following relationship:

5 $$\begin{aligned} v = \frac{r^2}{4\alpha t} \end{aligned}$$

From Eq. 5, the following expressions are obtained:

6 $$\begin{aligned} t = \frac{r^2}{4\alpha v} \quad \quad \quad\quad \frac{dt}{dv} = - \frac{r^2}{4 \alpha v^2} \end{aligned}$$

Eq. 5 and 6 can be used to rearrange the integral as follows:

7 $$\begin{aligned} T(x,y,t) = T_0+\frac{\Phi }{4 \pi \alpha \rho c_p}\int ^{\infty }_{u} \frac{1}{v}e^{-v} dv = T_0+\frac{\Phi }{4 \pi \alpha \rho c_p}E_I(u) \end{aligned}$$

where the variable $u = \frac{r^2}{4 \alpha t^*}$ and the function $E_I(u)$ is the exponential integral function. Expanding the solution to the actual physical variables *x*, *y*, and $t \equiv t^*$, the following final expression is obtained:

8 $$\begin{aligned} T(x,y,t) = T_0+\frac{\Phi }{4 \pi \alpha \rho c_p}E_I\left( \frac{(x-x_0)^2+ (y-y_0)^2}{4 \alpha t}\right) \end{aligned}$$

Several approximations of the exponential integral function $E_I$ are available in the literature; in this study we utilised an approximation^36,37^, which is often used in engineering applications :

9 $$\begin{aligned} E_I(u) = (A^{-7.7}+B)^{-0.13} \end{aligned}$$

where *A* and *B* have the following form:

10 $$\begin{aligned} \begin{array}{ll} A = ln [\left( \frac{0.56146}{u}+0.65\right) (1+u)]\\ B = u^4e^{7.7u}(2+u)^{3.7} \end{array} \end{aligned}$$

**Fig. 2:**
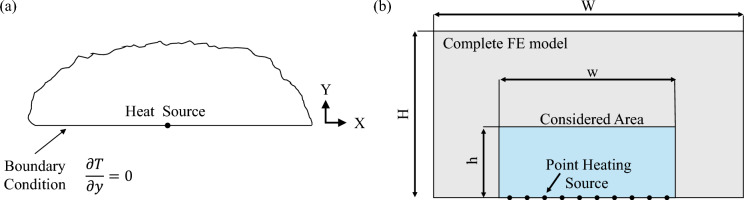
Schematic of a semi-infinite plate that represents a cross-section of the considered component: (**a**) analytical model with a point heat source located at the boundary, and (**b**) FE model with multiple point heat sources.

Using Eq. 9, the previously obtained solution of the problem becomes a fully analytical expression. Considering the presence of a semi-infinite plate with Neumann boundary condition, representing a vertical section of the investigated component, and the heat source located at the boundary, as depicted in Fig. 2a, we can assume that all the energy, radiated by the source, is reflected in the opposite direction. Based on these considerations, the following final analytical solution is obtained:

11 $$\begin{aligned} T(x,y,t) = T_0+\frac{\Phi }{2 \pi \alpha \rho c_p}E_I\left( \frac{(x-x_0)^2+ y^2}{4 \alpha t}\right) \end{aligned}$$

**Fig. 3:**
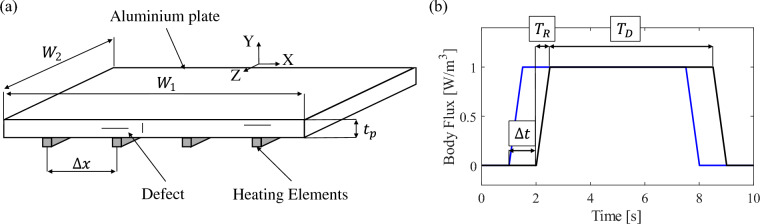
Schematic representation of the FE model of aluminium plate (**a**) and associated body flux activation law (**b**).

The proposed PAT utilises a thermal arrays, made of multiple point heat sources. By applying the superposition principle, assuming that the heating elements are equally spaced with distance $\Delta x$ and fired after a constant time $\Delta t$, two simple close-form analytical solutions can be obtained for the steering and for focusing of the thermal wave front. To steer the thermal wave and obtain an inclined wave front, contiguous thermal elements are fired in sequence along the desired direction. This results in the following closed-form solution:

12 $$\begin{aligned} T(x,y,t) = T_0+\frac{\Phi }{2 \pi \alpha c_p \rho } \sum _{p=1}^{N} H(t-\Delta t(p-1) ) E_I\left( \frac{(x-\Delta x (p-1) + \Delta x \ C)^2+ y^2}{4 \alpha (t-\Delta t(p-1) )}\right) \end{aligned}$$

where *H* is the Heaviside function, *p* is a counter, *N* is the number of heating elements, and *C* is the number of the heating element that represents the centre of the thermal array. To focus the wave front in a specific location, contiguous heating elements are fired from the extremities to the centre after intervals of time that scale quadratically, resulting in the following closed-form expression:

13 $$\begin{aligned} T(x,y,t) = T_0 + \frac{\Phi }{2 \pi \alpha c_p \rho } \sum _{p=1}^{N} H(t-\tilde{t}^2) E_I\left( \frac{(x-\Delta x (N-p-C))^2+ y^2}{4 \alpha (t -\tilde{t}^2)}\right) \end{aligned}$$

where $$\tilde{t} = \Delta t (|C-(N-1)/2|-|N-p-C|+(N-1)/2)$$.

### 2D finite element model

To validate the solution of the analytical model, an equivalent 2D FE model is modelled in Abaqus. To simulate a semi-infinite plate, a reduced portion (240 mm $\times$ 50 mm) of a large plate (1 m $\times$ 1 m) is considered in the FE analyses, as shown in Fig. 2b. The FE model is modelled with DS4 and DS3 thermal elements and presents Neumann boundary conditions along the entire external border. Using this approach, boundary effects are limited and the FE model simulates the behaviour of a semi-infinite plate. The thermal loads are simulated using concentrated heat flux loads of 64 W/m and are placed along one side of the border. The equivalent analytical model utilises the same dimensions, thermal input loads, and boundary conditions of the FE model which are reported in Table 1 for completeness.

**Table 1:** Dimension and thermal loads applied to the 2D FE and analytical models.

Quantity	H [mm]	W [mm]	h [mm]	w [mm]	N. Sources	Load [W/m]	$\Delta x$ [mm]	$\Delta t$ [s]
Value	1000	1000	50	240	21	64	10	0.1

### 3D finite element model

A 3D FE model representing an aluminium plate is utilised to perform numerical analyses and evaluate the effectiveness of the proposed PAT method. Fig. 3a shows a schematic representation of the considered FE model: the plate presents dimensions equal to 400 mm $\times$ 400 mm $\times$ 20 mm and several defects with different orientations. For the sake of simplicity, different types of defects are considered separately in the FE analyses, specifically, using three versions of the presented FE model:

1. Model 1: It presents a single horizontal defect with a constant width equal to 8 mm, passing through the middle of the plate at 10 mm from the surface
2. Model 2: It presents three inclined defects with planar dimensions 16 mm $\times$ 76 mm (D1 and D3) and 16 mm $\times$ 48 mm (D2), placed along a diagonal of the plate at different depths, namely 12 mm (D1), 6 mm (D2), and 2 mm (D3).
3. Model 3: It presents three flat bottom defects with radius equal to 35 mm (D1), 60 mm (D2), and 15 mm (D3), placed along a diagonal of the plate at different depths, namely 4 mm (D1), 10 mm (D2), and 16 mm (D3))

To simulate a crack, i.e. an infinitesimally thick defect, the defect is modelled by detaching nodes of adjacent elements and removing any form thermal contact transmission. Details of the geometry, dimension, and location of each defect are shown in Fig. S1 of the Supplementary Material. To obtain a continuous temperature distribution, 38 equally spaced heating elements are applied to the bottom surface of the aluminium plate. The elements presents a section area of 4 mm $\times$ 4 mm and cover the entire length of the plate. To replicate a real-case scenario, the FE model utilises Neumann and Robin boundary conditions which are mathematically represented by the following expressions:

14 $$\begin{aligned} \begin{array}{ll} \partial T(\textbf{x},t)/\partial n = 0 \\ \partial T(\textbf{x},t)/\partial n = -\frac{h}{k}(T(\textbf{x},t)-T_\infty ) \end{array} \end{aligned}$$

where *h* is the heat transfer coefficient, $T_\infty$ is the environmental temperature, and *n* is the selected direction for the derivative, e.g. *y*-direction.

**Table 2:** Dimensions, properties, and thermal loads applied to the 3D FE model.

Quant.	$W_1$ [mm]	$W_2$ [mm]	$t_p$ [mm]	N. Sour.	Load [W/m$^3$]	$h_L$ [W/(m$^2$K)]	$h_t$ [W/(m$^2$K)]
Value	400	400	20	38	4e6	100	300

**Table 3:** Material properties applied to the 3D FE model. Material properties are obtained from the following references^38–42^.

Sample	$\rho$ [kg/m$^3$]	*k* [W(m K)]	$c_p$ [J/(kg K)]
Aluminium (squared plate)	2700	237	897
Graphene (heating elements)	2200	4000	700

In the simulations it is assumed that the plate is placed on the ground and that it does not exchange heat flux with it. To this end, Neumann boundary conditions are applied to the bottom surface of the plate. The remaining surfaces, instead, utilise Robin boundary conditions to simulate the heat dissipation via convection with the surrounding air. Specifically, a room temperature of 288 K and two heat transfer coefficients ($h_L$ for the lateral surfaces and $h_t$ for the top surface of the plate) are applied to the FE model. Table 2 summarises the dimensions and the physical properties of the considered FE model while Table  3 reports the material properties that are used in the numerical simulations. In order to have simulations that are representative of both indoor and outdoor scenario, strong natural convection with air is considered in the numerical simulations. In the FEA, each heating element introduces a body flux of 4 $mW/mm^3$ to generate the desired thermal wave and perform the thermographic analysis. The body flux is calculated from an equivalent surface heat flux of 6080 $W/m^2$, a value typically used in PT^43^. To generate a steered thermal wave, the heating elements are activated in sequence, following the time law that is graphically represented in Fig. 3b. The delay between the activation of one heating element and the next one is indicated with the symbol $\Delta t$ while the time for which the element is constantly powered is denoted by $T_D$. A ramp activation function is used to activate and deactivate the heating elements, and it is denoted by the symbol $T_R$. By controlling $\Delta t$, $T_R$, and $T_D$ the desired wave front can be generated in the plate. To provide the same amount of energy in all the numerical experiments, the parameters $T_R$ and $T_D$ are fixed respectively at 0.5 s and 30 s while $\Delta t$ is modified to control the wave front direction. Finally, all the FE models utilises 3D diffusive heat transfer reduced element (DC3D8R) to model the aluminium plate and the heating elements.

**Table 4:** Measured resistance values of the nitinol wires that are installed in the composite/aluminium plates.

Arduino channel	A1	A2	A3	A4	A5	A6	A7	A8	A9	A10
Composite sample	11.5 $\Omega$	10.3 $\Omega$	11.1 $\Omega$	11.8 $\Omega$	11.0 $\Omega$	10.4 $\Omega$	13.3 $\Omega$	11.5 $\Omega$	12.3 $\Omega$	11.5 $\Omega$
Aluminium sample	8.7 $\Omega$	4.0 $\Omega$	5.23 $\Omega$	4.5 $\Omega$	5.9 $\Omega$	6.1 $\Omega$	4.4 $\Omega$	N/A	N/A	N/A

**Fig. 4:**
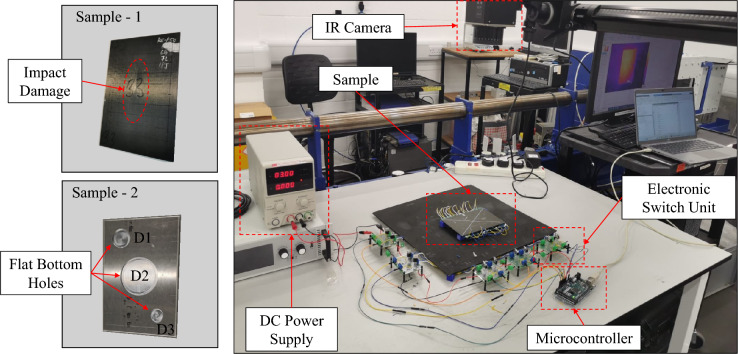
Experimental test rig and composite/aluminium samples.

## Experimental test rig

To validate the numerical models and test the efficacy of the proposed PAT method, a laboratory test rig for thermographic analyses is developed and used to apply PAT. The experimental set-up is shown in Fig. 4: an IR thermal camera (VarioCAM® HD Head) with a temperature resolution up to 0.05 K and accuracy of ± 1.5 K is used to capture thermographic images of the samples, using a frequency rate of 30 Hz. Two samples are experimentally tested: the first one is a composite plate with dimensions 150 mm $\times$ 100 mm $\times$ 2 mm that was damaged by high-energy impact testing at 18J. The second sample is an aluminium plate with dimensions 120 mm $\times$ 80 mm $\times$ 3 mm and with three flat bottom holes. The flat bottom holes are located along one diagonal of the plate and have the following diameters and depths: diameter = 20 mm and depth = 2.5 mm (D1), diameter = 40 mm and depth = 2 mm (D2) and diameter = 14 mm and depth = 0.5 mm (D3). Nitinol wires with a diameter of 0.1 mm and 0.2 mm are applied on the bottom surface of the samples and are used as heating elements in the experimental set-up. Specifically, the first sample utilises 10 equispaced nitinol wires ($\Delta x$ = 14 mm) with a diameter of 0.1 mm. The second sample, instead, uses 7 equispaced nitinol wires ($\Delta x$ = 18 mm) with a diameter of 0.2 mm. The temperature of the nitinol wires is controlled via Joule effect: two DC source controllers (max output 30 V / 10 A) are utilised to impose a controlled voltage on the nitinol wires which are connected in parallel via electronic switch units. A microcontroller (Arduino Mega 2560) is used to control the gates of MOSFET that are installed in the electronic switch: by opening/closing the MOSFET gates at specific time instants, the required input control law is applied to the heating elements and the associated steered/focused wave is induced in the analysed samples. Tab.4 shows the experimental values of the resistance of the nitinol wires for the two analysed sample plates. Although the wires are similar, differences in the length and the presence of imperfections in their shapes and connections results in different values of resistance. The experimental plate samples and the test-rig of Fig. 4 are utilised to conduct experimental analyses. PAT is performed by controlling the heating elements and imposing a constant voltage of 3V for analysing the composite plate and a constant voltage of 11V for analysing the aluminium plate. The composite plate has a lower internal conductivity, thus thermal gradients are maintained for longer periods in the component. This enables the sample to be exposed to the external heat source for longer periods and permits PAT to use larger $\Delta t$. In addition, the composite plate sample utilises nitinol wires with a diameter of 0.1 mm which increases the resistance of each heating element. This results in the necessity of a reduced amount of power ($\approx$ 8 W) to perform the experimental analyses. On the contrary the aluminium plate sample has a larger conductivity and is embedded with nitinol wires with larger diameters, thus it requires a larger amount of power ($\approx$ 160 W) to perform the experimental tests. This enables similar variations in surface temperature across both experimental tests, allowing for a direct comparison of the experimental results.

## Results

This section introduces the results of the study: firstly, the results of the analytical model are presented and compared against thermographic images of an equivalent 2D FE model; then, the numerical results of a 3D FE model of an aluminium plate are introduced and presented. Finally, the experimental results, obtained with the composite and aluminium plate samples, are shown. Post-processing techniques, such as absolute temperature contrast^2,44^, are utilised to visualise the numerical and experimental thermographic data.

**Fig. 5:**
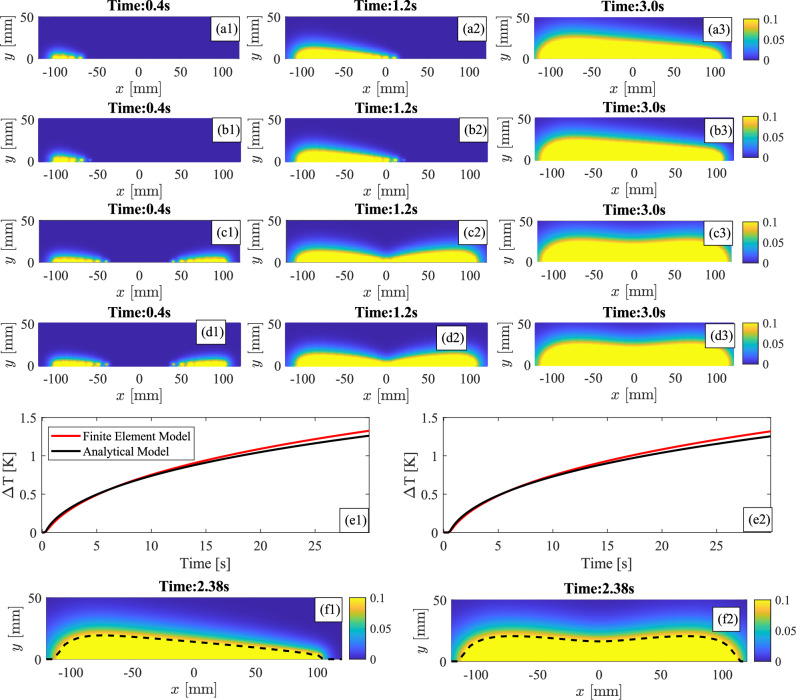
Comparison between $\Delta T$ distributions (limited between 0 and 0.1 K) at different time instants in the considered section areas for the analytical model and FE model. All the temperatures are plotted as temperature differences.

### Analytical solutions

Analytical and numerical FE simulations are performed to evaluate the evolution of thermal wave-front during wave steering and wave focusing in a semi-infinite cross section of an aluminium plate. In all the simulations the thermal loads are activated in sequence after a $\Delta t$ time and are kept active for all the simulation time (about 30 s). To visualise the thermal wave, the scale is limited between 0 - 0.1 K and the temperatures are expressed as temperature differences $\Delta T$ with respect to a general initial temperature $T_0$.

**Fig. 6:**
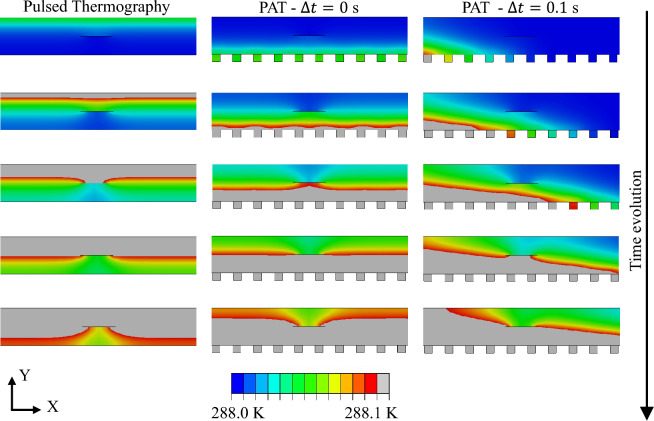
Thermal wave propagation in the central cross section (Plane XY at Z = 200 mm) of the aluminium plate with an horizontal crack during PT (left column), PAT with $\Delta t = 0$ s (central column), and PAT with $\Delta t = 0.1$ s (right column). Five time instants during the passage of the wave front through the defect are shown.

**Fig. 7:**
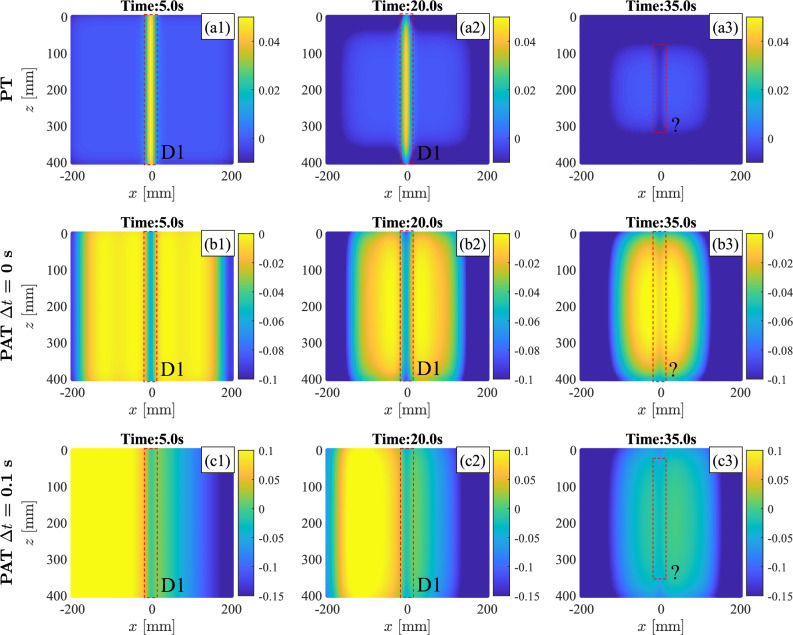
Comparison between PAT and PT using the same input power. The thermographic images of the top surface are obtained using the 3D FE model with a passing-through horizontal defect and the post-processing technique of temperature contrast. The reference point for the contrast is set at X = 40 mm and Z = 200 mm.

**Fig. 8:**
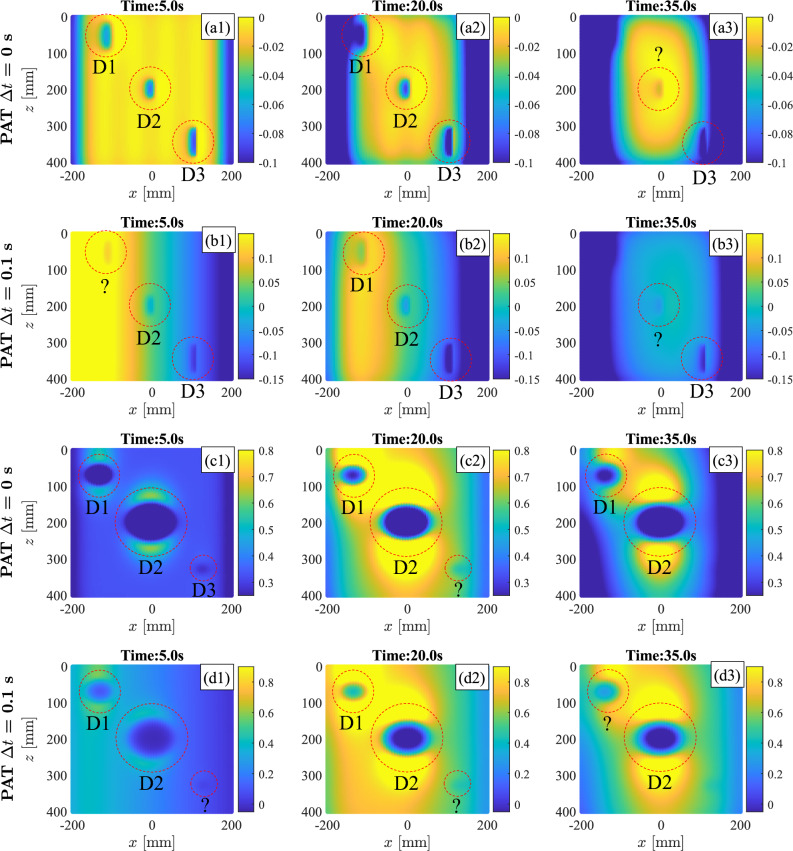
Thermographic images of the top surface of the 3D FE model featuring inclined defects (**a**1–**b**3) and flat bottom holes (**c**1-**d**3). Thermography is performed using PAT with $\Delta t = 0$ (**a**1–**a**3, **c**1–**c**3), and PAT with $\Delta t = 0.1$ s (**b**1–**b**3, **d**1-**d**3). Three time instants at 5.0 s, 20.0 s, and 35.0 s are considered. Data are visualised with the post-processing technique of temperature contrast with reference point at X = 40 mm and Z = 200 mm.

**Fig. 9:**
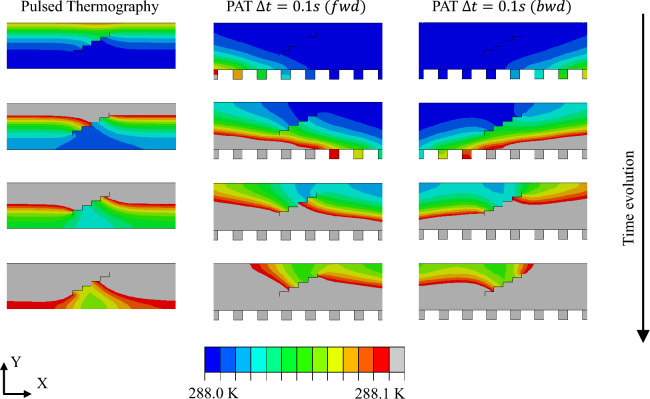
Thermal wave propagation in the central cross section (Z = 200 mm) of the aluminium plate with inclined defects during PT (left column), forward PAT with $\Delta t = 0.1$ s (central column), and backward PAT with $\Delta t = 0.1$ s (right column). Four time instants during the passage of the wave front through the defect are shown.

**Fig. 10:**
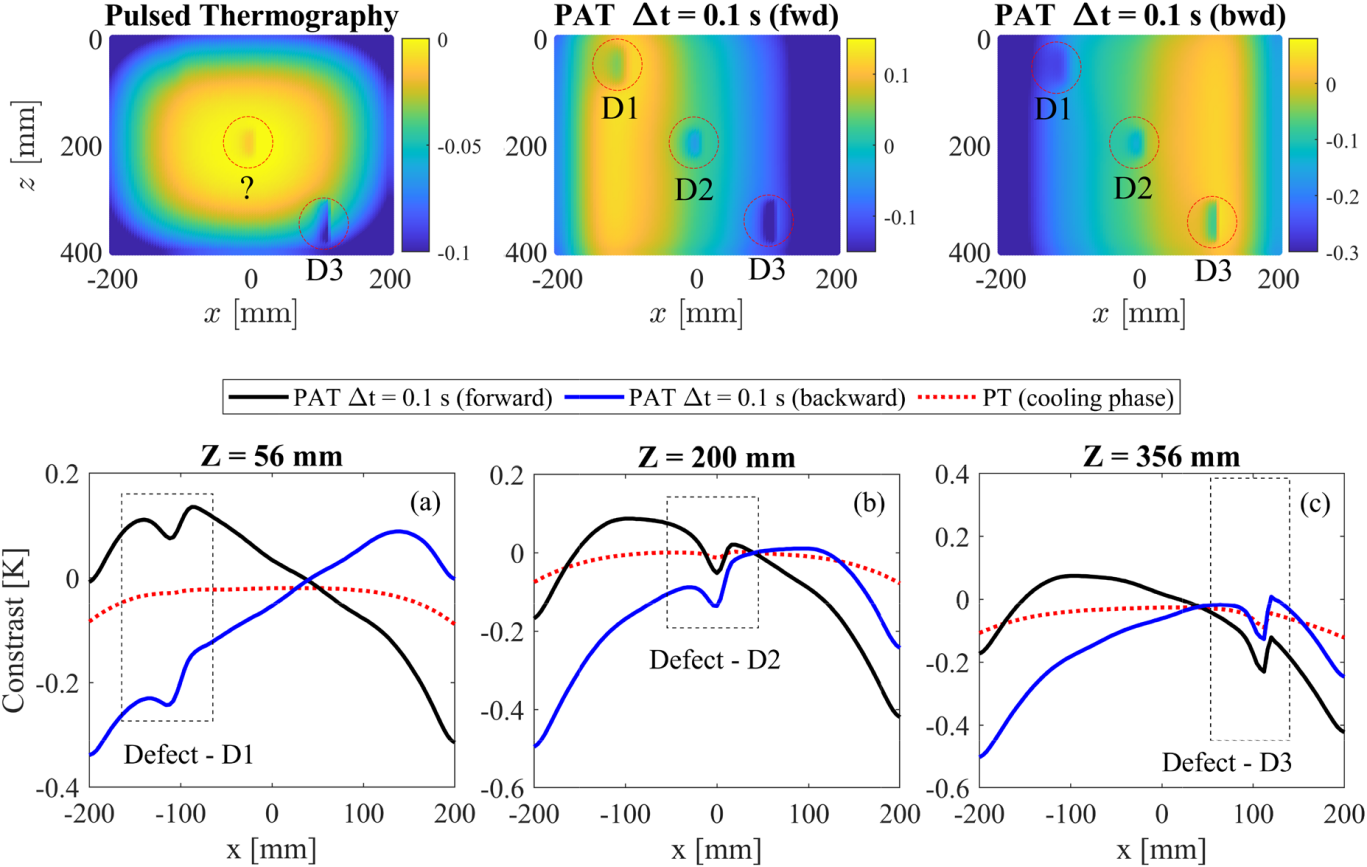
Thermographic images (upper panels) and contrast distributions along direction x (lower panels) of the aluminium plate with inclined defects. PAT are obtained at t = 20 s (heating phase) while PT thermograms are obtained at t = 55 s (cooling phase). The contrast distribution are illustrated in correspondence of the three defects selecting a time frame with large contrast for each case. Both PT and PAT utilised the equivalent input thermal power. Data are visualised with the post-processing technique of temperature contrast with reference point at X = 40 mm and Z = 200 mm.

Figure 5a1–d3 show the progression of the thermal wave front in the semi-infinite cross-section for 3 seconds of simulation: the panels in the first row (Fig. 5a1–a3) show the temperature distributions that are obtained with the analytical model when wave steering is performed while the panels on the third row (Fig. 5c1–c3) show the temperature distributions of the same model when wave focusing is performed. Equivalent simulations for wave steering and wave focusing are performed using the 2D FE model and the results are reported in Fig. 5b1–b3 and Fig. 5d1–d3. The results show that the temperature distribution of the analytical model is very close the temperature distribution of the FE model, for all the considered time instants. In addition, the temperature time evolution of a single point, located at X = -50 mm and Z = 5 mm, of the analytical and the equivalent FE model is evaluated and compared in Fig. 5e1 for wave steering and in Fig. 5e2 for wave focusing. The results show that time histories of the analytical and numerical models follow similar trajectories.

The analytical solutions for wave steering/focusing of Eqs. 12 and 13 are used to obtain the previously introduced results. Nonetheless, they represent the forward solution of the thermodynamic problem, thus several iterations might be required to identify the actual wave front for a certain $\Delta x$ and $\Delta t$. To circumvent this problem, we propose to utilise the inverse solution of the problem which can directly reconstruct the fully developed thermal wave front. Closed-form expression of the inverse solutions for wave steering and wave focusing are not achievable, nonetheless Eqs. 12 and  13 can be used to obtain robust numerical solutions. The problem reads:

15 $$\begin{aligned} Z_{y} = T_1 - T(X_v, t_v,y) \end{aligned}$$

where $T_1$ is the reference temperature at which the thermal wave is visualised, $T(X_v, t_v,y)$ is the forward solution of the analytical model at a prescribed time $t_v$ for a prescribed vector $X_v$ of horizontal coordinates, and $Z_{y}$ is the quantity that must be minimised to solve the problem. Eq. 15 is numerically solved using the Levenberg-Marquard scheme implemented in the MATLAB function fsolve() and considering the closed-form solutions of Eqs. 12 and 13. The results are reported in the bottom panels of Fig. 5 where the temperature distribution of the plate is compared with the directly computed thermal wave front (dashed black line). Specifically, Fig. 5f1 shows the wave front for wave steering at 2.38 s while Fig. 5f2 shows the wave front for wave focusing at the same time instant. The panels, together with the additional results of Fig. S2, show that numerical solution of the inverse problem is able to accurately calculate the wave-front at a specific time instant for both wave steering and wave focusing.

### Finite element analysis

The previously introduced 3D FE models are utilised to perform an in-depth numerical investigation of the interaction between the generated thermal waves and the defects. For sake of brevity the analyses are limited to the steering case, but similar results are expected for wave focusing. The interaction between wave front and defect and the consequent effects on the thermographic images are analysed using 2D and 3D thermal images. PT is an established method in NDT and thermography: it demonstrated to be a reliable approach to identify defects that are parallel to heated surface, i.e. perpendicular to the generated heat flux (see for example the following references^45–47^), such as holes, large material defects, and material thickness reductions. For this reason, the PT analyses are used as reference for evaluating the performance of the proposed PAT. To enable a fair comparison, the same input power is utilised in PT and PAT simulations. Figure 6 shows the thermal distribution of a cross-section of the aluminium plate with an horizontal passing defect (FE model 1) at Z = 200 mm when PT and PAT are used. To visualise the wave front and its evolution in time, the scale is limited between 288 K (room temperature) and 288.1 K. This enables the visualisation of the propagation of the wave front across the thickness of the plate and its interaction with an horizontal defect of 8 mm. Three different cases are considered, namely PT (left column of Fig. 6), PAT with $\Delta t$ = 0 s (central column of Fig. 6), and PAT with $\Delta t$ = 0.1 s (right column of Fig. 6). The interaction between the defect and the thermal wave front results into the generation of a local gradient right behind the defect which can be captured by thermographic images of the upper surface of the component. In the case PAT with $\Delta t$ = 0 s, the local gradient distribution behind the defect is practically equivalent to the one obtained with PT, with the exception that the thermal wave propagates in the inverse direction. This difference arises from the heating method used in the two techniques: PT heats the upper surface of the component while PAT utilises heating elements that are applied on the bottom surface of the component. When PAT is used with a $\Delta t > 0$, the thermal wave is steered in the desired direction and its interaction with the defect generates an inclined and less strong gradient behind the defect.

The associated thermographic images of the top surfaces are reported in Fig.7 using absolute contrast temperature as post-processing technique: the panels in the first row show the results for PT, the panels in the second row show the results for PAT with $\Delta t$ = 0 s, and the last row show the results for PAT with $\Delta t$ = 0.1 s. The results in the panels of the first two columns of Fig.7 are obtained during the heating phase while the panels in the last column show the thermographic images during the cooling phase. Higher values of the contrast are obtained during heating phase: specifically the PT at t = 5 s is able to clearly identify the presence of the horizontal defect. Similar results are obtained with PAT with $\Delta t$ = 0 s which clearly identify the presence of the passing-through horizontal defect. In this case, the only difference from PT consists in the presence of temperature inhomogeneities at the boundaries of the plate, due to the lack of heating elements at X=-200 mm and X = 200 mm. Unlike the other two cases, PAT with $\Delta t = 0.1$ s presents a thermal wave that does not propagates perpendicularly to the heated surface, but rather laterally, acting as a sort of thermal scanning on the component and identifying substrate defects. In this case, PAT is able to clearly identify the presence and the location of the horizontal passing-through defect, especially during the heating phase. A three-dimensional representation of the thermographic images that are obtained with PAT with $\Delta t$ = 0 s and $\Delta t$ = 0.1 s is provided in the supplementary material in Figs. S3 and  S4. The figures demonstrate that the three-dimensional representation allows for a better detection of the passing-through horizontal defect which is clearly visible as a deep and sudden decrement of the surface representing the contrast temperature of the thermographic images. This is better highlighted in the temperature contrast distributions of Fig. S3a2,b2,c3 and S4a2,b2,c3 that are obtained by cutting the surface at Z = 200 mm. There, the defect is identified by a sudden local drop of temperature contrast. The figures show that the local drop is constantly maintained during the heating phase; nonetheless, as the simulation progresses, the visibility of the drop diminishes due to the increasing temperature gradient at the borders.

PAT analyses are repeated for 3D FE models featuring inclined defects (FE Model 2) and flat bottom defects (FE Model 3). The results are reported in Fig. 8 when $\Delta t$ = 0 s and $\Delta t$ = 0.1 s are used. The panels in the first two columns show the heating phase of the process while the panels in the last column show the cooling phase of PAT. The PAT is able to detect the presence of all the considered defects, clearly identifying their borders and their locations. When no steering is performed, i.e. with $\Delta t$ = 0 s, the incidence between the propagating thermal wave and the defects is maximised and results in a better identification of the flaws, as shown by Fig. 8a1–a3,c1–c3. Higher contrast is found at the beginning of the heating phase (time instant t = 5.0 s) for PAT with $\Delta t$ = 0 s. When $\Delta t > 0$, the PAT acts as “scanner” and defects are better identified at later stages of the process. In the Supplementary Material, Fig. S5 and S6 show the three-dimensional representation of the numerical thermographic results of PAT with $\Delta t$ = 0.1 s when, respectively, three inclined defects and three flat bottom holes are considered in the FE models. Temperature contrast trends along the X-direction are obtained in correspondence of the defects and are shown in the panels (a2-c2) of the figures. The contrast trends demonstrates that the defects can be identified as sudden and localised drops in the thermographic results. Larger and less deep defects, such as D1 and D2 in the flat bottom holes model and D3 in the model with inclined defects, result in larger contrast drops which consequently lead to an easier defect detection in a 2D thermographic image. On the contrary, smaller defects, such as D3 in the model with flat bottom holes, are difficult to identify using the classical 2D thermographic representation of Fig. 8 while instead they result to be clearly visible using the 3D approach shown in the Supplementary Material in Fig. S6.

To show the importance of the wave incidence in the detection of inclined defects, the interaction between the inclined defects and steered waves is investigated in detail using FEA. To this end, PT and PAT at $\Delta t = 0.1$ s are utilised to perform the analysis of FE model 2, with PAT firing the heating elements in the forward (as shown in Fig, 8b1-b3) and backward direction. The thermal wave propagation and interaction with the central defect is shown in Fig. 9. PT generates a perpendicular thermal wave from top to bottom that interacts with the defect in a single way while PAT has different wave-defect interactions depending on the steering angle: in the forward case, the wake weakly interacts with the defect, generating a small thermal gradient behind the defect; on the contrary, the backward case creates a thermal wave that strongly interacts with defect, inducing a strong temperature gradient. The resulting thermograms and temperature contrast distributions are shown in Fig. 10: thermographic images with PAT are capture at t = 20 s, during the heating phase, while thermograms of PT are obtained at t = 55 s, during the cooling phase. Forward and backward PAT, thanks to their scanning nature, are able to locate all three defects while PT is able to detect only flaws that are close to the surface, such as D3. As the selection of contrast scale may affect the detection of defects, a quantitative comparison between PAT and PT is carried out by comparing temperature contrast trends at z = 56 mm, z = 200 mm, and z = 356 mm, where defects are located. The trends are illustrated in the bottom panels of Fig. 10 and are selected for time instants that maximise the contrast of the defect for each case. The results show that PAT has larger contrast than PT for the deep defect D1 (z = 56 mm), the middle defect D2 (z = 200 mm), and the surface defect D3 (z = 356 mm). Finally, the stronger interaction between the wave and defects in the backward case, results into slightly deeper drops of the temperature contrast trends in correspondence of the defects, especially for defects D3 and D2 which are firstly met by the steered thermal wave.

### Experimental analysis

**Fig. 11:**
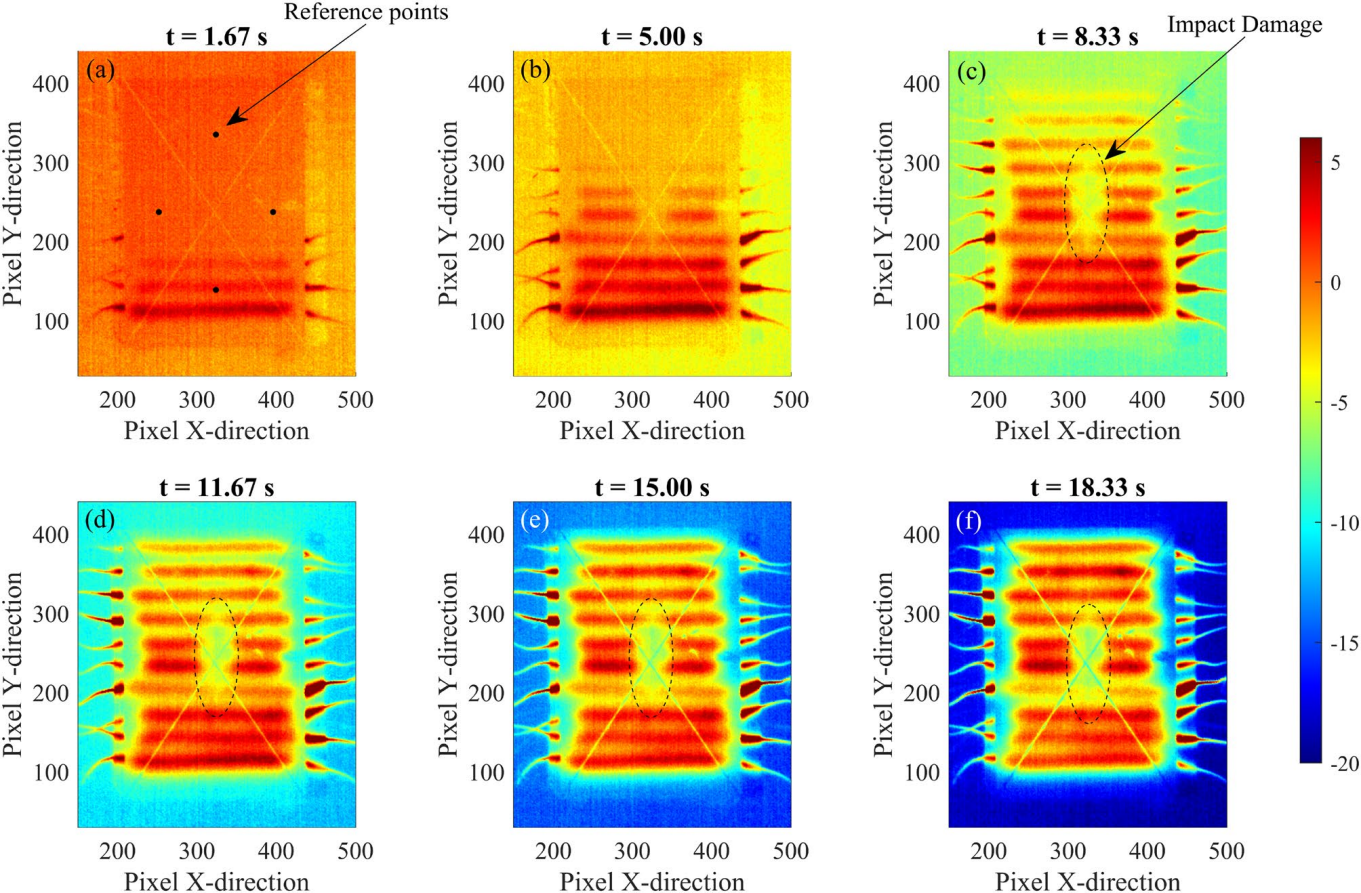
Thermographic images of a composite plate with an impact damage. PAT is applied imposing a $\Delta t = 1$ s and a total activation time of 40 s. Thermograms are obtained in terms of contrast-to-noise ratio using the reference points of panel (**a**).

**Fig. 12:**
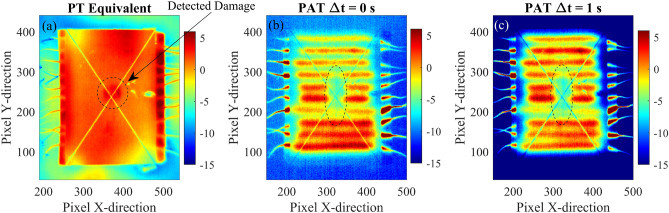
Comparison between equivalent PT (**a**), PAT with $\Delta t$ = 0 s (**b**), and PAT with $\Delta t$ = 1 s (**c**) of the composite plate. The figure shows thermographic images in terms of contrast-to-noise ratio where contrast is obtained using reference points as indicated in Fig. 11(**a**).

**Fig. 13:**
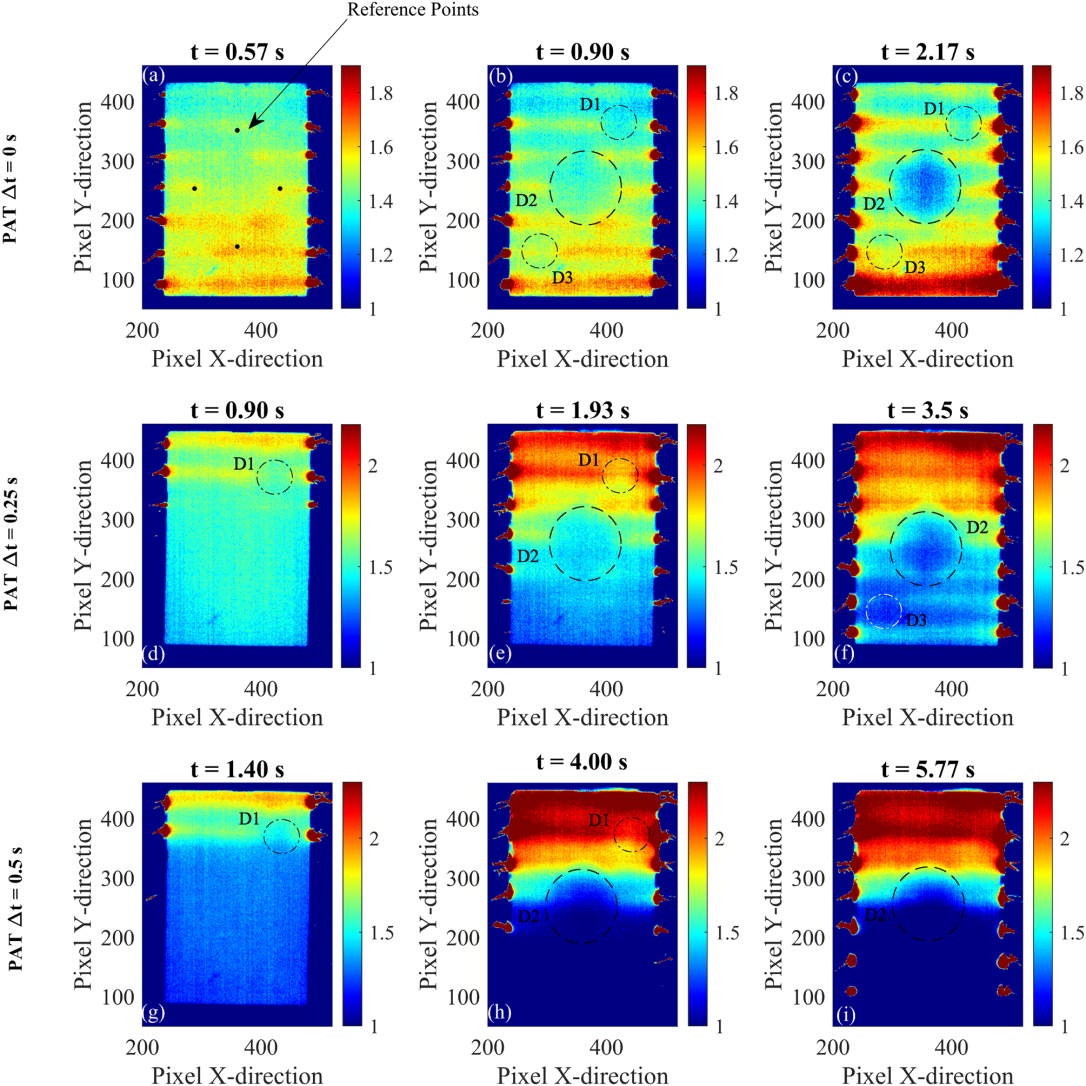
Thermographic images of a aluminium plate with three flat bottom holes. PAT is applied imposing a $\Delta t = 0$s, $\Delta t = 0.25$s, and $\Delta t = 0.5$s for a total activation time of 5 s. The figure shows thermographic images in terms of contrast-to-noise ratio where contrast is obtained using reference points of panel (**a**) .

**Fig. 14:**
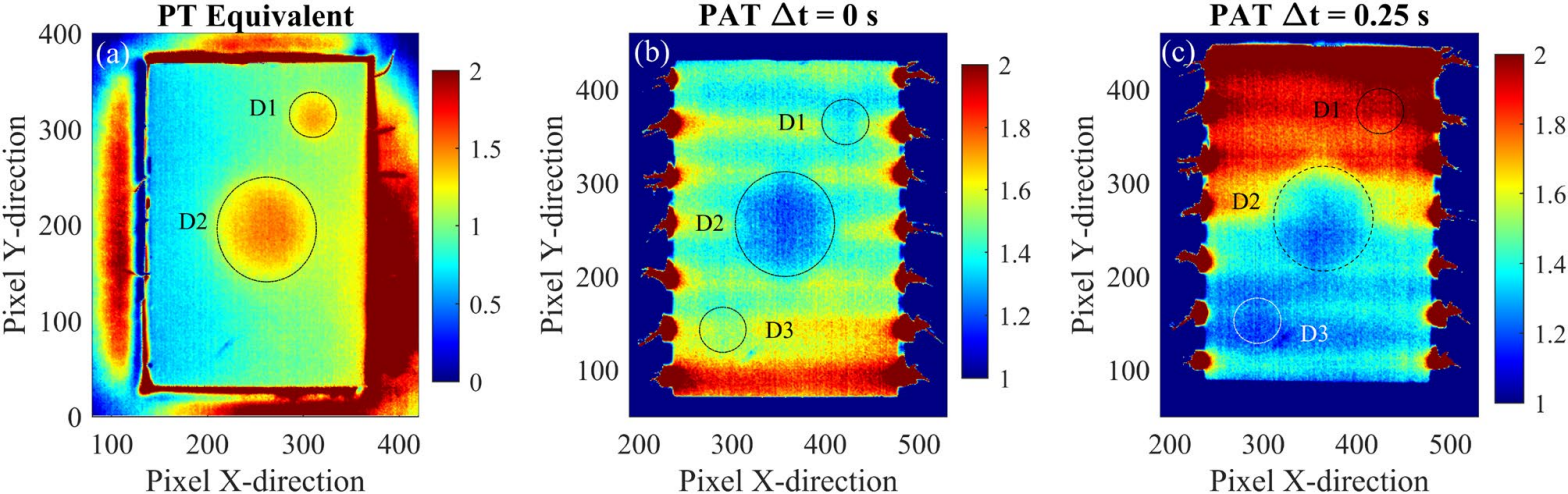
Comparison between equivalent PT (**a**), PAT with $\Delta t$ = 0 s (**b**), and PAT with $\Delta t$ = 1 s (**c**) of the aluminium plate. The figure shows thermographic images in terms of contrast-to-noise ratio where contrast is obtained using reference points as indicated in Fig. 13(**a**).

Experimental thermographic images of the composite and aluminium plates are obtained using the previously introduced test-rig in terms of Contrast-to-Noise-Ratio (CNR). To this end, the classic Signal-to-Noise Ratio (SNR) for thermographic analysis (see for example^48^ and reference therein) is revised as follows:

16 $$\begin{aligned} CNR = (C_s(x,y) -\mu _n)/\sigma _n \end{aligned}$$

where $C_s(x,y)$ is the contrast temperature, $\mu _n$ is the mean of the noise, and $\sigma _n$ is the standard deviation. The mean and the standard deviation of the measurement is obtained from the initial thermogram of the sequence, before the heating sources are activated. The results are reported in Figs. 11 and 12, for the composite plate, and in Figs. 13 and 14, for the aluminium plate. Figure 11 shows the 2D thermographic images obtained with the post-processing technique of temperature contrast and PAT with $\Delta t$ = 1 s. Specifically, temperature contrast is obtained by using the mean of the temperature of the reference points illustrated in Fig. 11a. The figure shows several time instants of the heating phase and illustrates the scanning effect of the proposed method. During the initial phase of the thermographic analysis, the defect is not identified as the thermal gradient and the associated thermal wave have not interacted yet with the defect. From $t > 8$ s, the defect becomes clearly visible as shown by panels (c-f) of Fig. 11.

Experimental thermography of the composite plate is also performed using a PT equivalent procedure: the procedure consists in heating the upper surface of the sample with a heat gun (Steinel 3520, max power 1800 W) and monitor the associated temperature change. The plate is firstly heated homogenously and then thermographic images are captured, using the IR thermal camera. The results are shown in Fig. 12 along with thermographic images of the composite plate obtained with PAT with $\Delta t =0$ and $\Delta t =1$ s. The figure shows that the equivalent PT, although using a large thermal power, is able to locate only small portion of the defect which is close to impact region. The analyses performed with PAT instead shows the presence of a more extended temperature anomaly which incorporates the delamination of the layers of composite material in the surrounding area of the impact. Fig. 12c shows that, using a time delay of 1 s in PAT, it is possible to clearly locate the defect and the extension of the delamination of the impact damage, as the inclined gradient is able to interact with defect and to compensate possible inhomogeneities in the induced temperature distribution of the plate. Such inhomogeneities are typically found in experimental tests and are due to imperfections in the experimental set-up, e.g. different resistances of the nitinol wires.

Figures  13 and 14 show the experimental thermographic images of the aluminium plate sample in term of CNR. The thermographic images are obtained using the proposed PAT with $\Delta t$ = 0 s (panels (a-c)), $\Delta t$ = 0.25 s (panels (d-f)), and $\Delta t$ = 0.5 s (panels (g-i)) and three different time instants of the heating phase are shown. The simultaneous activation of all the heating elements allows for the three defects to be clearly identified in 2D thermographic images, especially after a few seconds from the activation of the heating elements. When the time delay $\Delta t$ is set to 0.5 s, the resulting thermal wave is strongly steered and the interaction with the defects is limited, resulting in the identification of only the defects D2 and D1, as shown by panels (g-i) of Fig. 13. When $\Delta t$ is set equal to 0.25 s, PAT is able to locate all the three defects, especially defect D3 which results in a darker region in the bottom of the plate, as shown in Fig. 13f. Experimental three-dimensional thermographic images and CNR temperature trends along the aluminium plate for PAT with $\Delta t$ = 0 s and $\Delta t = 0.25$ s are shown in Fig. S7 and Fig. S8 of the Supplementary Material. The images are filtered with a 2D Gaussian filter ($\sigma = 5$) to eliminate the presence the noise introduced by the thermal camera and the environment during the experimental testing. To this end, the MATLAB function imgaussfilt() is used to post-processed the data. Similarly to the numerical analyses, the experimental temperature trends are obtained by cutting the 3D thermographic representation at specific X positions along the Y-direction in correspondence of the three defects. Differently from the numerical analyses, the experimental analyses are conducted with a reduced number of heating elements (only seven for the aluminium plate) which results in local temperature fluctuations, as shown in Fig. S7 a2,b2,c2 and Fig. S8 a2,b2,c2. Although the presence of temperature fluctuations, the figures demonstrate that the three-dimensional thermographic images and CNR trends result in an easier and faster detection of the defects compared to the classical 2D thermography, even in experimental testing. To further compare the proposed PAT with classical PT, the equivalent PT procedure is also applied on the aluminium plate. The results are shown in Fig. 14 where equivalent PT and PAT are compared. The 2D thermographic images, presented in terms of contrast-to-noise ratio, show that proposed PAT is able to locate all the three defects. On the other hand, the equivalent PT detects the presence of larger defects such D2 and D1 but it is not able to locate small and shallow defects that are not close to the surface, such as the defect D3.

## Discussion and conclusions

This paper proposes a novel active IRT technique, named Phased-Array Thermography (PAT), for the accurate detection and location of defects/damages in aerospace and mechanical components. The method exploits several controlled heating elements which works as a thermal phased-array and controls the direction of propagation of the induced thermal wave in the evaluated component. A new post-processing approach of thermographic data, based on a three-dimensional representation, is proposed and utilised to detect temperature anomalies and defects in the investigated components. Firstly an analytical model for the prediction of wave steering and wave focusing is proposed and validated against numerical simulations. Then, finite elements simulations of an aluminium plate with several defects are performed to evaluate PAT and compare its detection capabilities with widely used IRT techniques, such as Pulsed Thermography (PT). Finally, a composite plate characterised by an impact damage and an aluminium plate with flat bottom holes are experimentally analysed via PAT.

The analytical models proposed in Sect. Analytical model are tested and validated in Sect. Analytical solutions, where the numerical and analytical results are presented and qualitatively compared. The results demonstrate that, by controlling the parameters $\Delta t$ and $\Delta x$, it is possible to control to the thermal wave front, generating a steered or a focused thermal wave, as shown in Figs. 5 and S2. All the presented diagrams demonstrate the remarkable similarity between the numerical and the analytical model, with only small differences after long simulation times. Nonetheless, as with any analytical model, it is important to remember that there are limitations to its applicability. Indeed, the wave steering (Eq. 12) and wave focusing (Eq. 13) models are derived under the assumptions of a semi-infinite plate and heat point sources. The first assumptions hold only if boundary effects and thermal wave reflection do not play an important role in the thermodynamics of the system. Therefore, the model can be utilised to determine the orientation of the thermal wave in the around of the heating sources. The second assumption, instead, holds when heating sources are well separated and sufficiently small with respect to the dimension of the considered plate. Thus, the model is suitable to evaluate the effect of small and localised heat sources, like the one that are utilised in the proposed FE analyses and in the experimental testing. The proposed analyses and validations demonstrate that the analytical model is capable of identifying the induced thermal wave in a cross-section of the investigated component with reduced computational burden. This makes the analytical model an effective and valuable tool for designing the thermal arrays in PAT, especially in the early design stages. However, given the limitations, accurate FEA with 3D modelling should be considered for finalising the design of the thermal array.

The finite elements model of an aluminium plate and the associated analyses are presented in Sect. 3D finite element model. Firstly it is demonstrated how the induced thermal wave interacts with the presence of a defect. This thermodynamic mechanism is illustrated in Fig. 6 and in Fig. 9 where it is shown that thermal waves that are perpendicular to the defect induce stronger and more localised temperature gradient anomalies behind the flaw. Such anomalies can be captured by a IR camera, detecting the presence of the defects. While currently available IRT procedures, such as PT, can only induce temperature gradients perpendicular to the heated component surface, the proposed PAT has the ability to control the direction and the shape of the induced thermal waves. Thus, PAT can induce stronger temperature anomalies behind defects that are not oriented in the same direction of the surface and for which other IRT techniques would have a limited effect. More importantly, PAT is not limited to generation of perpendicular thermal waves but it can produce several steered and focused waves and, through the combinations of the results, it can provide clear picture of the flaws scenario in the investigated component. This claim is supported by the thermographic numerical results of Figs.7, 8, and 10, where the presence of horizontal passing-through, inclined, and flat bottom hole defects is detected using PAT. The results demonstrate that PAT with $\Delta t$ = 0 s, i.e. synchronous activation of the heating elements, produced 2D thermographic images that are comparable and very similar to the ones obtained using PT during the heating phase. The results also shows, that a clearer identification of the defects is obtained during the heating phase in both PAT with $\Delta t$ = 0 s and PT. This represents an advantage for the proposed procedure, as thermographic images obtained PT can be only captured during the cooling phase when the lamps are off. When $\Delta t > 0$ , PAT acts a sort of “scanner” as inclined thermal waves are induced and they travel from one side to the other of the investigated component. This allows PAT to detect the presence of the defects that are oriented in any direction. In fact, the inclined thermal wave interacts in a different way with defects, depending on their orientation, increasing or decreasing the gradient anomaly behind the defects. This concept is clearly shown in Figs. 9 and  10: when PAT with $\Delta t = 0.1$s is tested against inclined defects firing the thermal array in the forward direction, the gradient anomaly is slightly reduced, because the thermal waves are oriented along the direction of propagation of the inclined cracks, resulting in less effective thermographic images of the defects. On the contrary, when the direction of propagation is inverted, the wave strongly interacts with the defect, enhancing the defects detection via thermographic analysis. Similarly, as shown by Fig. 8c1-d3, the thermal wave strongly interacts with flat bottom holes only when the heating elements are fired simultaneously, i.e. when the wave is directed perpendicularly to the defects.

The three-dimensional representation of the thermographic data offers a novel approach to the post-processing of thermography. Results for numerical analyses of a plate with an horizontal passing-through defect, inclined defects, and flat bottom holes are reported in Figs. S3,  S4,  S5, and  S6 of the Supplementary Material. Using this approach defects are located and identified in a easiest and faster way than the classical 2D approach, as they appear as sudden drops in the temperature/contrast distribution. By using the three-dimensional post-processing approach, the scale adopted in the thermographic images becomes less important and even small defects, far away from the component surface, are located and detected. For example, Fig. S5 demonstrates that the inclined defect D1, a deep defect, is easily detected and recognised with 3D thermographic images and temperature distribution along the X direction. Similarly, the defect D3, the deepest and smaller defect of the model with flat bottom holes, is promptly detected with the proposed 3D approach as shown in Fig. S6. It is worth notice, that the same defects are not visible using the 2D thermographic images of Fig. 8 as defect with higher contrast cover their presence.

To demonstrate the robustness and flexibility, the proposed PAT method is validated via experimental testing using two different samples with different defects, wire number, and wire diameter. To guarantee a fair comparison between the experimental analyses, the voltage of the external power supply was set to 3V during the analysis of the composite plate and 11V for the investigation of the aluminium plate. This allows the two components to achieve similar surface temperature. In the experimental set-up, several practical constrains limited the number of nitinol wires that are controlled. Firstly, large number of connections are difficult to handle and secondly, each nitinol wire requires a dedicated electronic switch unit to activate the current flow and control the thermal wave. Therefore, ten and seven nitinol elements are utilised in the experimental set-up of, respectively, the first and second sample. The number of elements and their distance are important parameters in the design of thermal array and in PAT, as they affect the thermal wave and its homogeneity. Thermal arrays with many close elements develop homogenous waves with flat wave front, especially near the heating elements. On the contrary thermal arrays with fewer and sparse elements generate less homogenous waves, characterised by fluctuation along the array. This influences the defect detectability of PAT, especially of those that are close to the thermal array. The distance between the elements can also affect the maximum steering angle and the quality of the steered thermal wave: for example, sparse elements increase the parameter $\Delta x$ of the thermal array and thus require large $\Delta t$ to achieve the same steering angle of thermal arrays that presents more elements. The experimental campaign considered these practical limitations, thus small samples with large defects were chosen to overcome these difficulties. The experimental results presented in Sect. Experimental analysis demonstrated the feasibility and the accuracy of the proposed PAT in detecting damages in real mechanical components, confirming the results of the numerical analysis. In particular, it is demonstrated that the PAT is able to accurately detect the extension of an impact damage in a composite plate, including the delamination of composite layers, as shown in Fig. 11 and Fig. 12. On the contrary, the equivalent PT is not able to determine the extension of the delamination, as thermographic images show the presence of only the region of impact. The experimental analysis of an aluminium plate with flat bottom holes generated similar results. The results are also post-processed using the proposed three-dimensional approach and are shown in Figs. S7 and  S8 of the Supplementary Material: while the presence of a reduced number of heating elements generate unwanted temperature fluctuations, the 3D post-processing approach produce good results even in the case of experimental thermographic images, detecting also small and deep defects such as the defect D3.

In conclusion, a novel IRT procedure, named PAT, is presented and analytically, numerically, and experimentally investigated. This novel method advances the field of non-destructive testing by introducing the concept of focused and steered thermal waves which enable a faster and a more accurate detection of flaws than classical approaches. Its demonstrated effectiveness, across different materials and defect types, provides a solid foundation for both industrial deployment and broader application beyond traditional NDT domains, where localised temperature concentration or steered wave-front are necessary.

## Supplementary Information

Supplementary Information 1.

Supplementary Information 2.

## Data Availability

The experimental raw data generated and analysed during this study and the input files of the FEA are included in this published article and its supplementary information files.
