# Supplementary material for: A novel phased-array thermography concept for non-destructive testing
Source: Sci Rep. 2025 Nov 7;15:39144. doi: 10.1038/s41598-025-26380-8 (PMC12594793; doi:10.1038/s41598-025-26380-8)
Supplement: Supplementary file 1 — Supplementary Information 1. [file 41598_2025_26380_MOESM1_ESM.pdf]

Supplementary Material

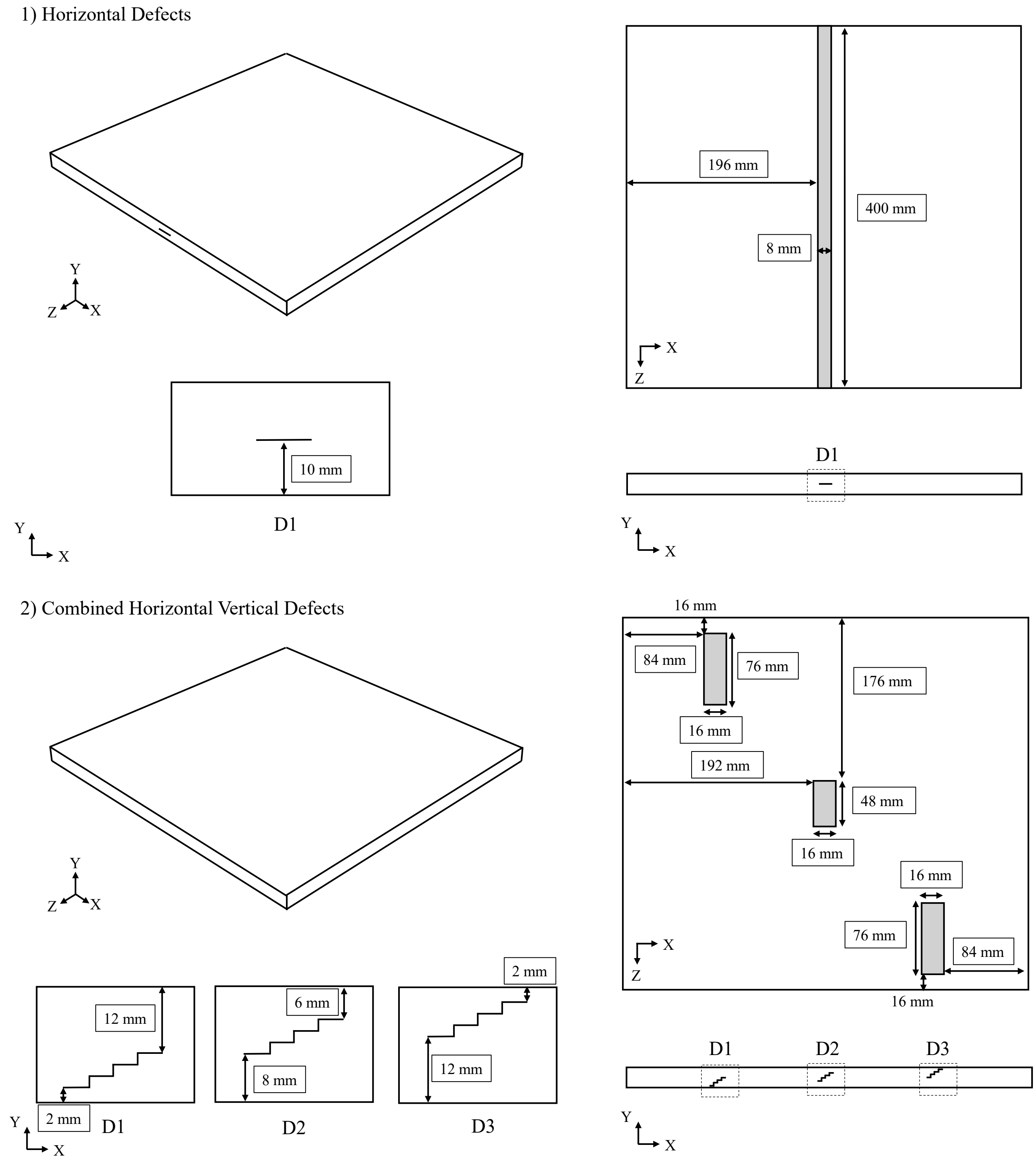

Figure S1. Geometry, dimension, and location of the three different types of defects that are considered in the FEA.

3) Flat Bottom Holes Defects

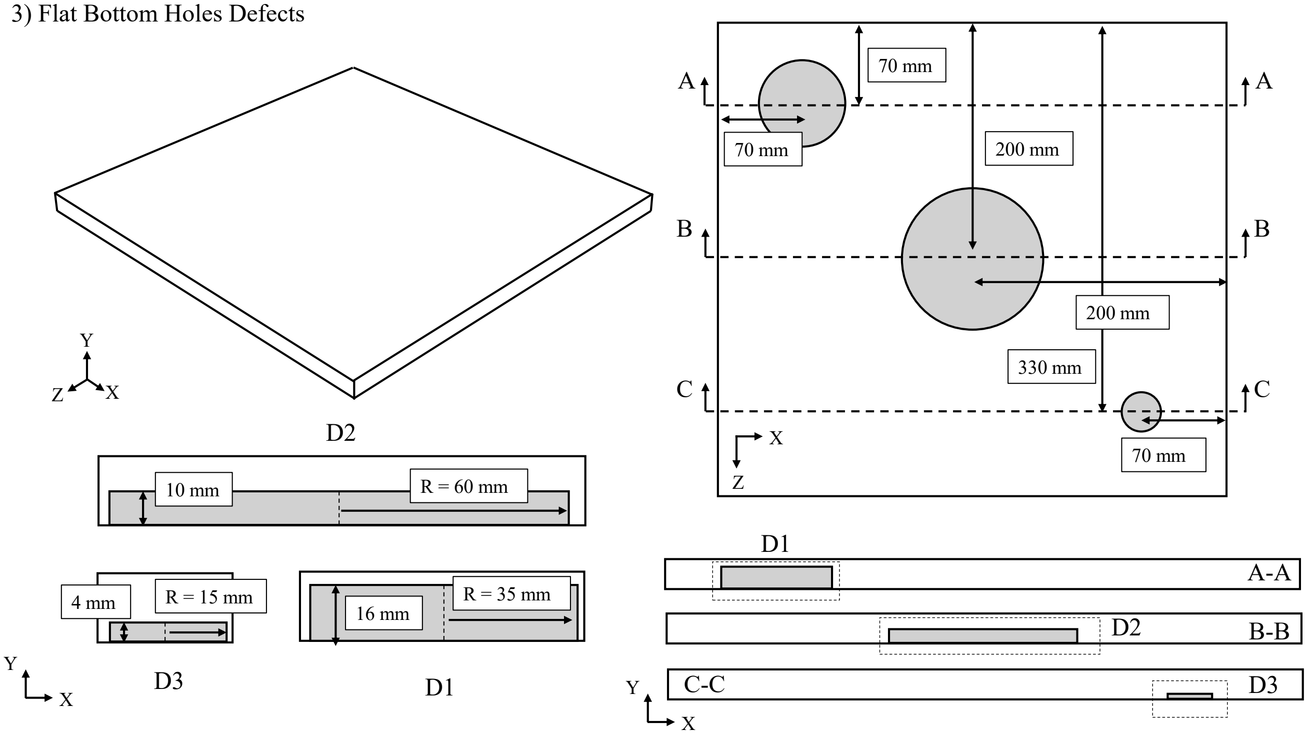

Figure S1. Geometry, dimension, and location of the three different types of defects that are considered in the FEA.

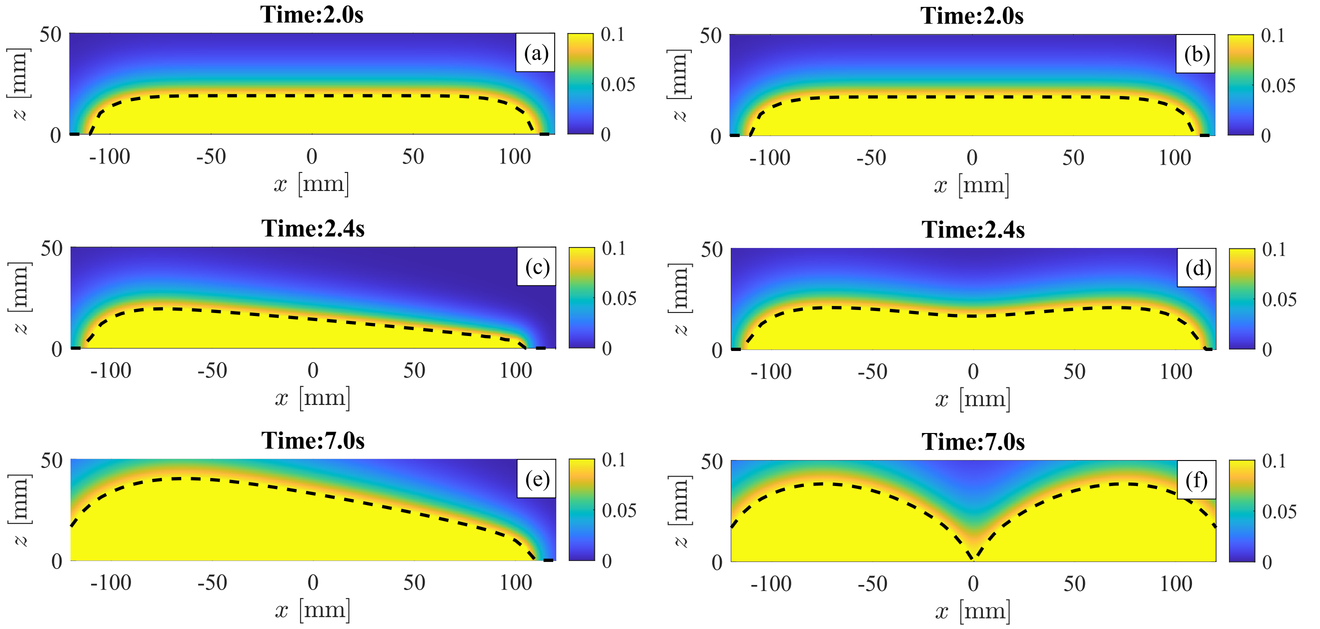

Figure S2. Thermal wave front identified via numerical solution of inverse problem at $\Delta T = 0.1$ K for wave steering (a,c,e) and wave focusing (b,d,f). Different time delays are utilised, namely $\Delta t = 0$ s (a,b), $\Delta t = 0.1$ s (c,d), and $\Delta t = 0.3$ s (e,f).

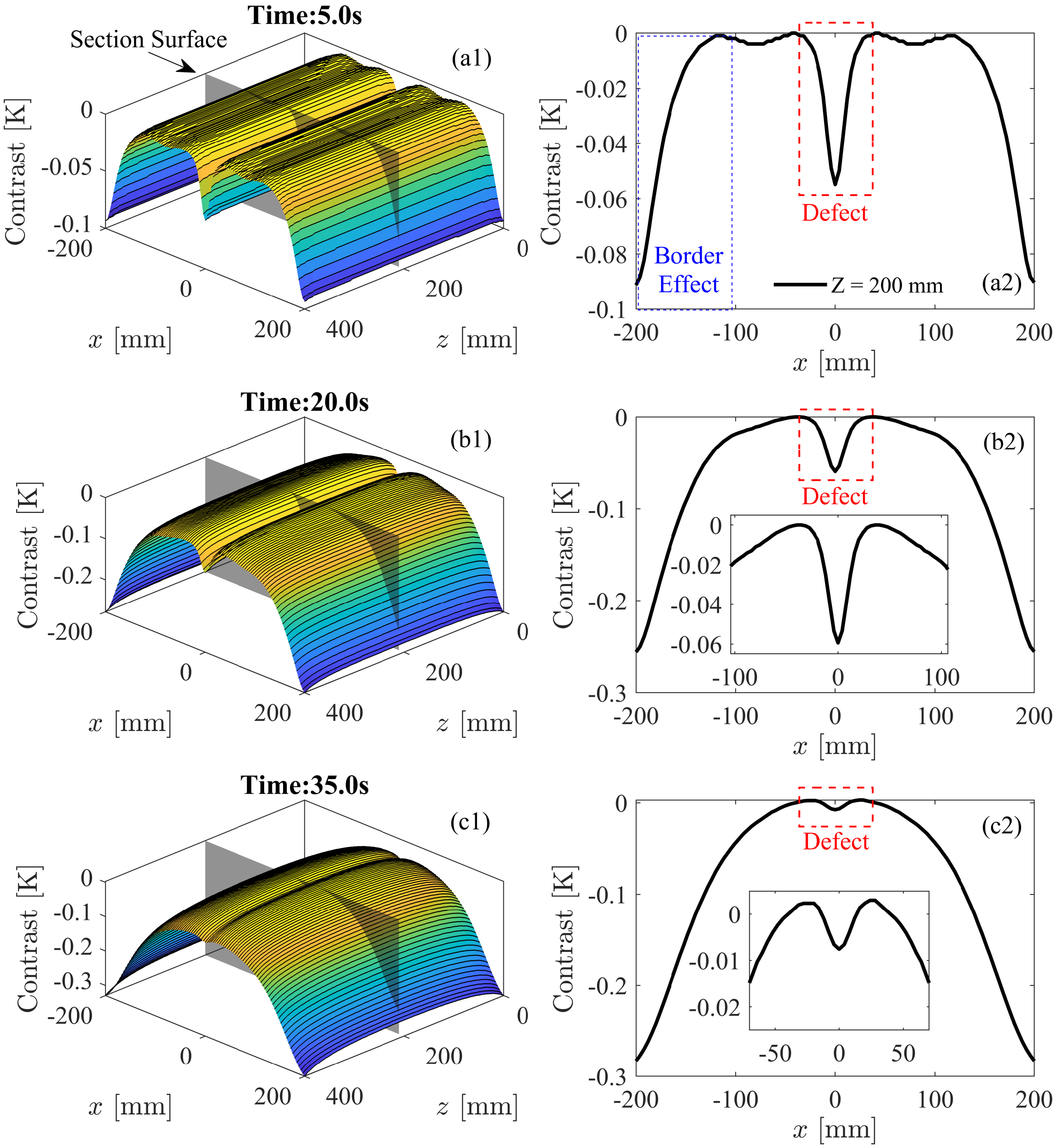

Figure S3. Three-dimensional representations and temperature distributions at Z = 200 mm of the thermographic images represented in Fig. 7(b1-b3). Thermography is obtained with PAT with $\Delta t = 0$ s considering the presence of a passing-through horizontal defect.

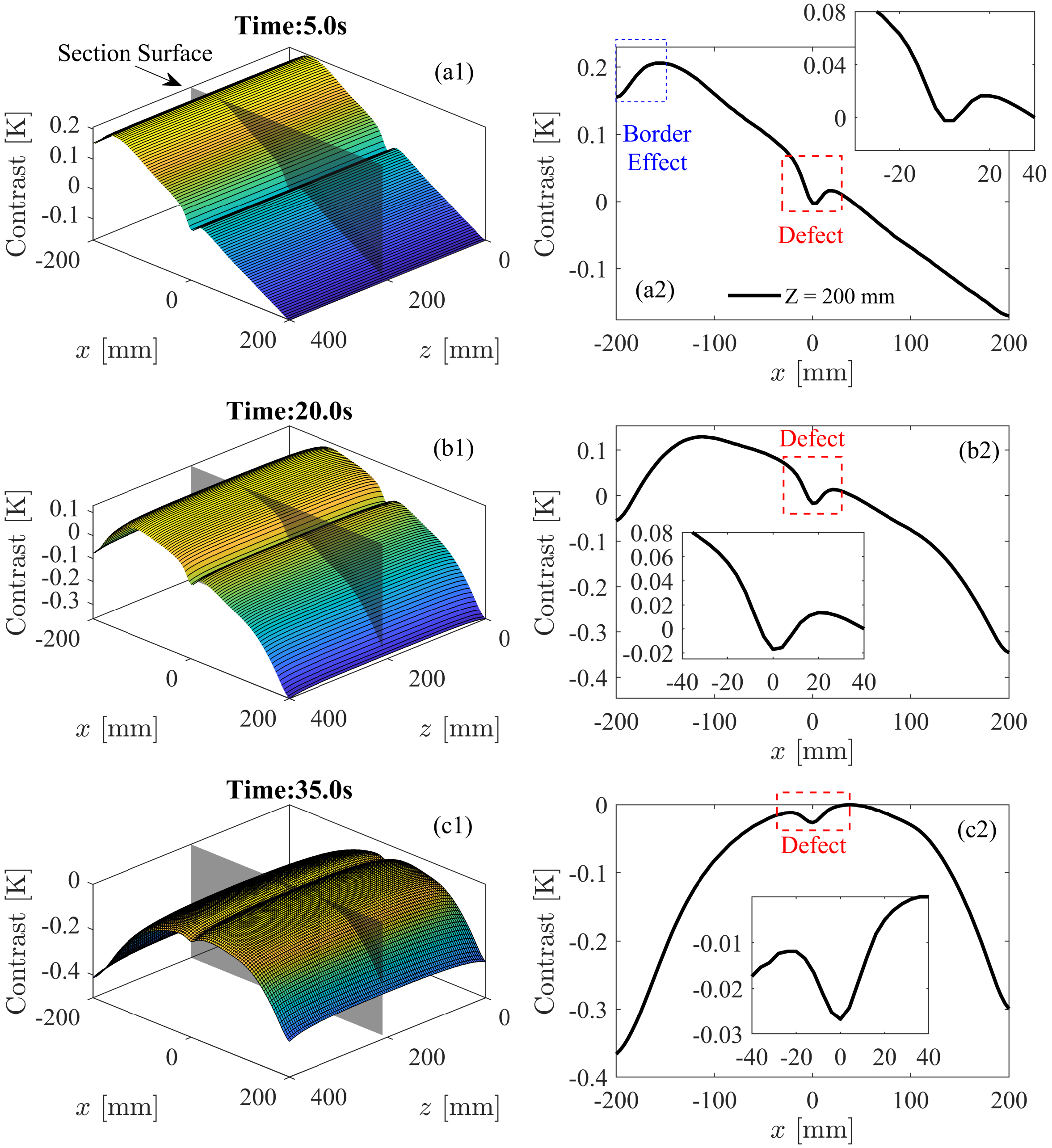

Figure S4. Three-dimensional representations and temperature distributions at Z = 200 mm of the thermographic images represented in Fig. 7(c1-c3). Thermography is obtained with PAT with Δt = 0.1 s considering the presence of a passing-through horizontal defect.

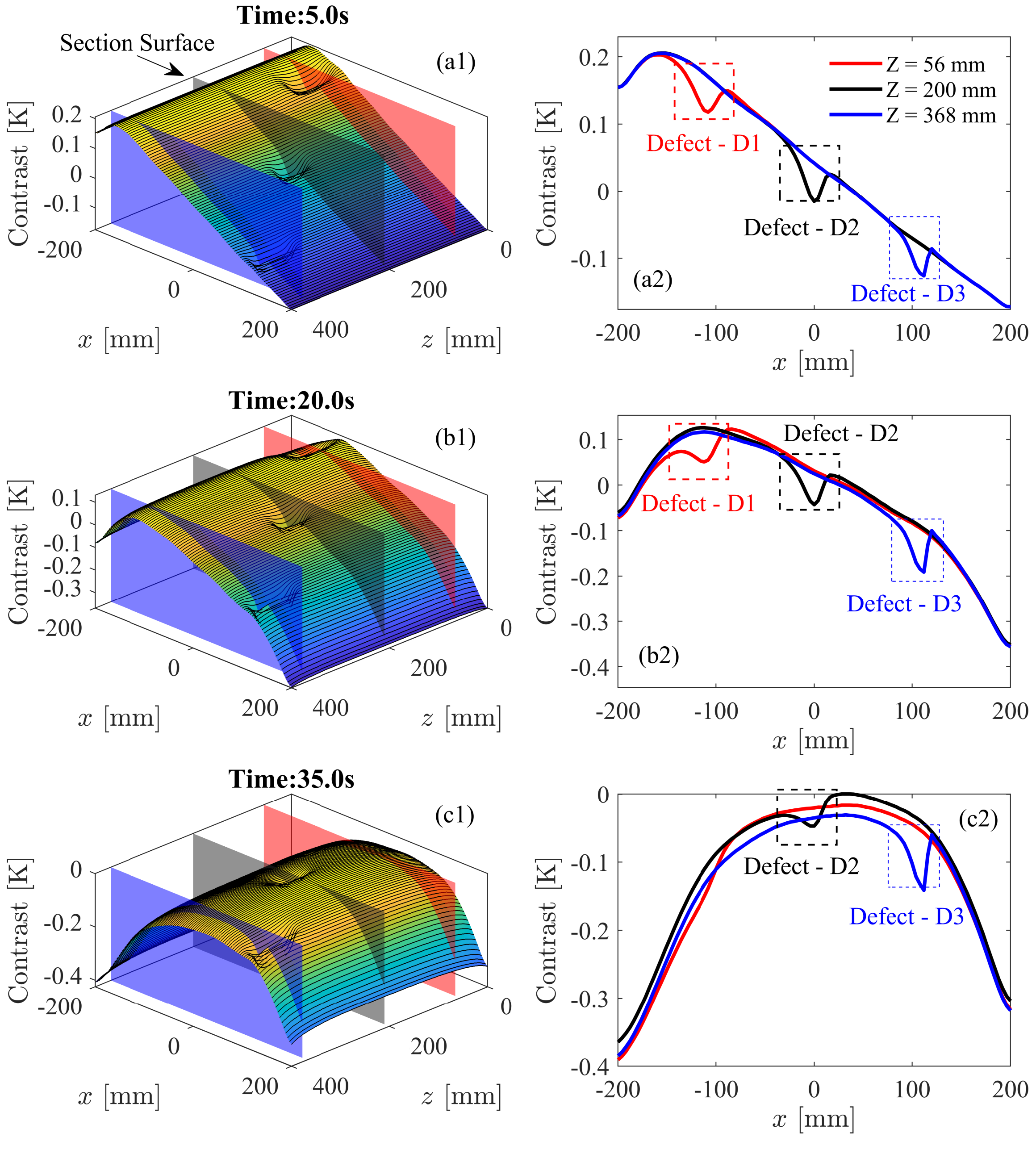

Figure S5. Three-dimensional representations and temperature distributions at Z = 56 mm, Z = 200 mm, and Z =368 mm of the numerical thermographic images represented in Fig. 8(b1-b3). Thermography is obtained with PAT with Δt = 0.1 s considering the presence of three inclined defects.

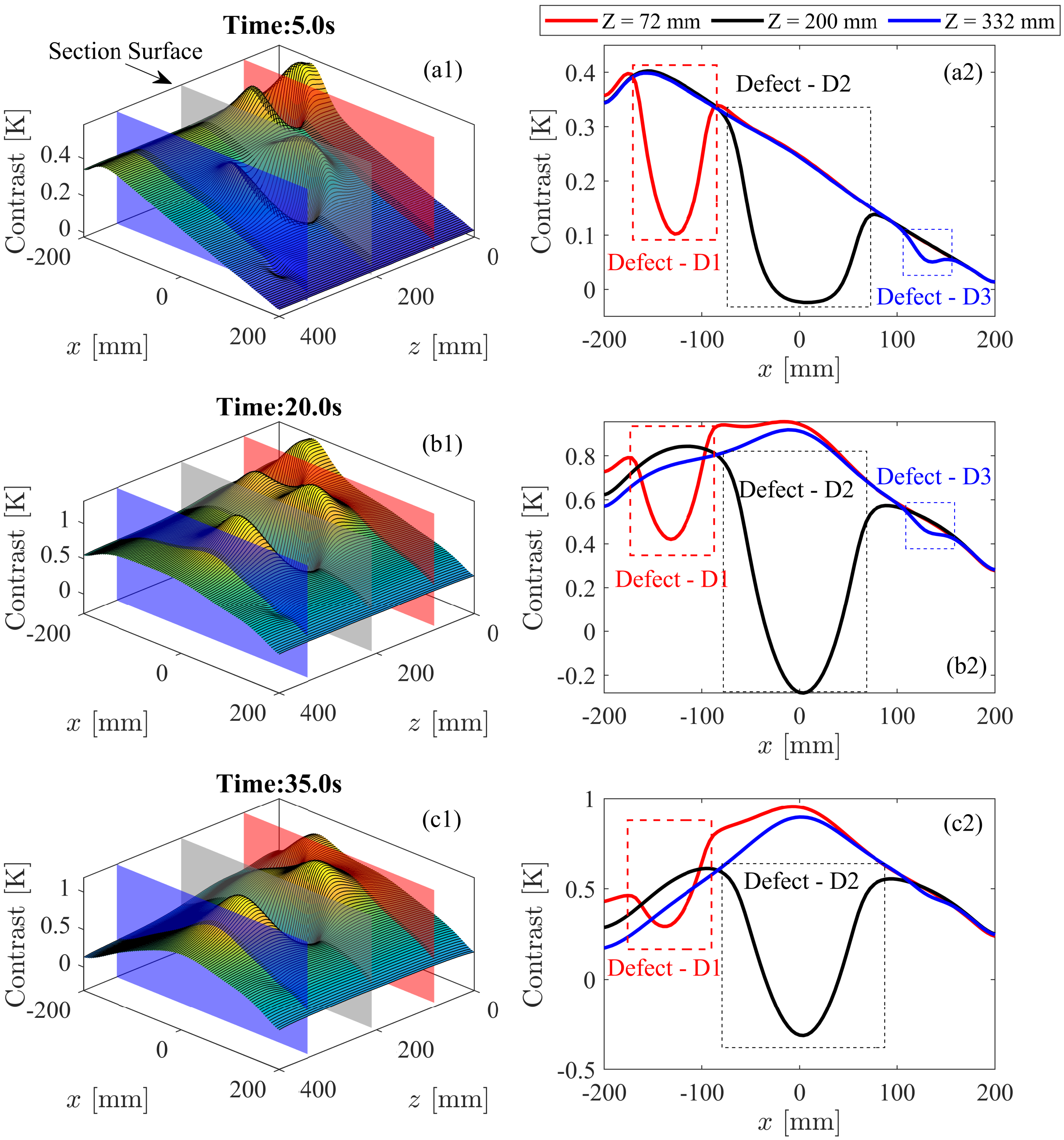

Figure S6. Three-dimensional representations and temperature distributions at Z = 72 mm, Z = 200 mm, and Z = 332 mm of the numerical thermographic images represented in Fig. 8(d1-d3). Thermography is obtained with PAT with $\Delta t = 0.1$ s considering the presence of three flat bottom holes.

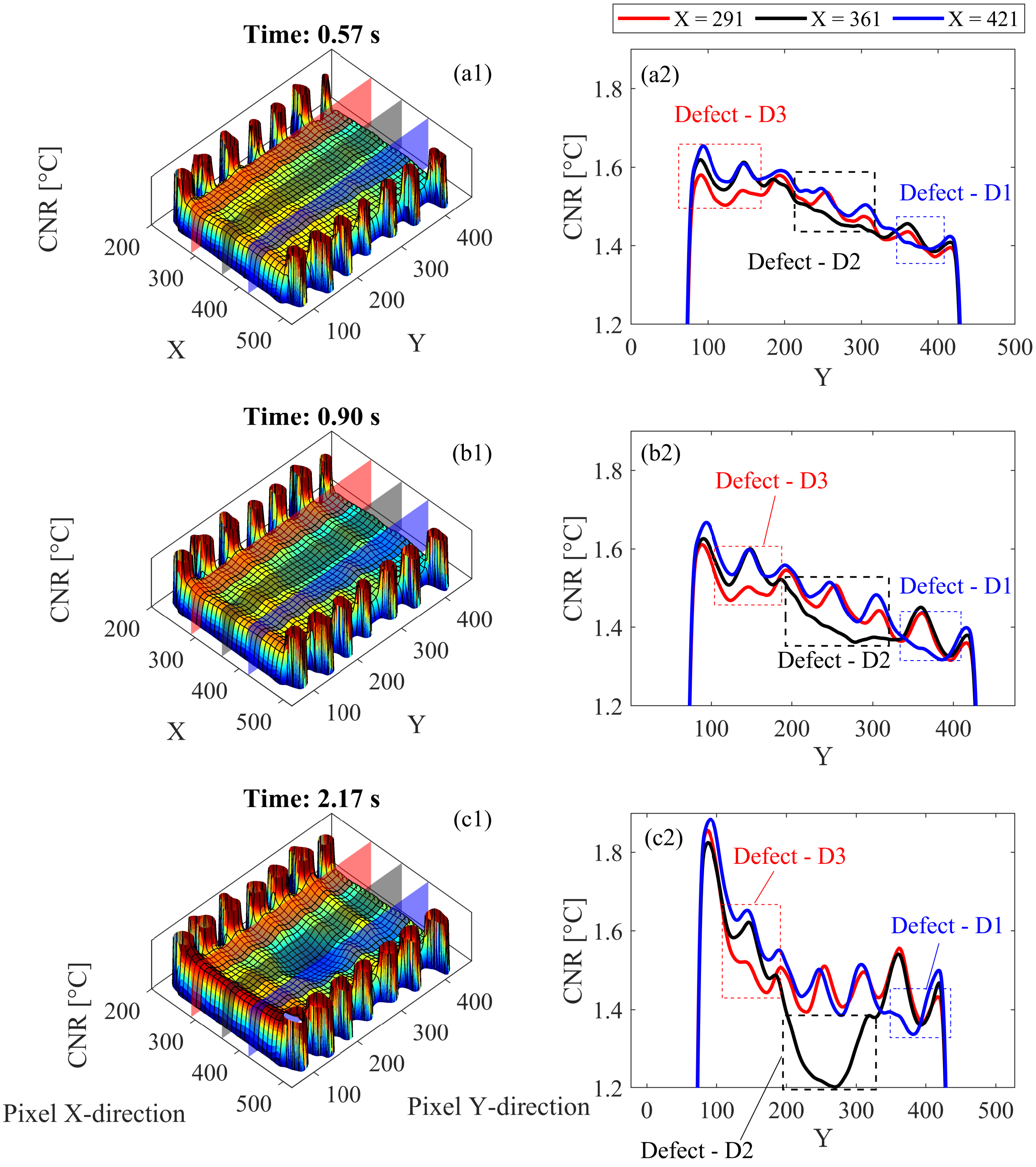

Figure S7. Three-dimensional representations and CNR distributions at X = 291 pixels, X = 361 pixels, and X = 421 pixels of the experimental thermographic images represented in Fig. 13(a-c). Thermography is obtained with PAT with $\Delta t = 0$ s using the aluminium plate sample which features the presence of three flat bottom holes.

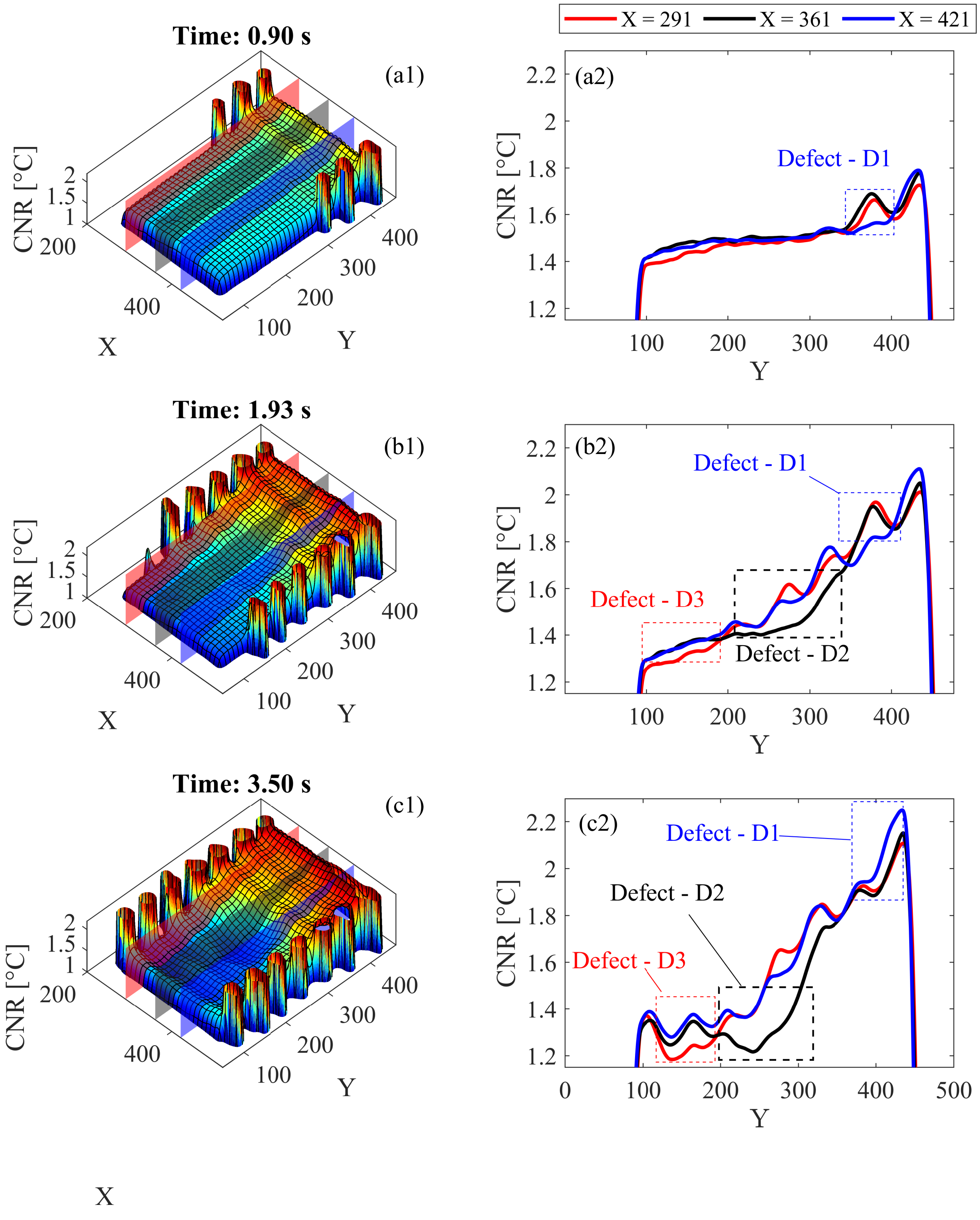

Figure S8. Three-dimensional representations and CNR distributions at X = 291 pixels, X = 361 pixels, and X = 421 pixels of the experimental thermographic images represented in Fig. 13(d-f). Thermography is obtained with PAT with Δt = 0.25 s using the aluminium plate sample which features the presence of three flat bottom holes.
